# *De novo* phosphatidylcholine synthesis is required for autophagosome membrane formation and maintenance during autophagy

**DOI:** 10.1080/15548627.2019.1659608

**Published:** 2019-09-13

**Authors:** Gabriela Andrejeva, Sharon Gowan, Gigin Lin, Anne-Christine LF Wong Te Fong, Elham Shamsaei, Harry G. Parkes, James Mui, Florence I. Raynaud, Yasmin Asad, Gema Vizcay-Barrena, Joanna Nikitorowicz-Buniak, Melanie Valenti, Louise Howell, Roland A. Fleck, Lesley-Ann Martin, Vladimir Kirkin, Martin O. Leach, Yuen-Li Chung

**Affiliations:** a Cancer Research UK Cancer Imaging Centre, Division of Radiotherapy and Imaging, The Institute of Cancer Research London and Royal Marsden Hospital, London, UK; b Cancer Research UK Centre for Cancer Therapeutics, The Institute of Cancer Research London, London, UK; c Department of Medical Imaging and Intervention, Chang Gung Memorial Hospital at Linkou, College of Medicine, Chang Gung University, Taoyuan, Taiwan; d Centre for Ultrastructural Imaging, King’s College London, London, UK; e Breast Cancer Research, The Institute of Cancer Research London, London, UK; f Molecular Pathology, The Institute of Cancer Research London, London, UK

**Keywords:** Autophagosome, autophagy, choline phospholipids, CTP:phosphocholine cytidylyltransferase, phosphatidylcholine, propargylcholine

## Abstract

Macroautophagy/autophagy can enable cancer cells to withstand cellular stress and maintain bioenergetic homeostasis by sequestering cellular components into newly formed double-membrane vesicles destined for lysosomal degradation, potentially affecting the efficacy of anti-cancer treatments. Using ^13^C-labeled choline and ^13^C-magnetic resonance spectroscopy and western blotting, we show increased *de novo* choline phospholipid (ChoPL) production and activation of PCYT1A (phosphate cytidylyltransferase 1, choline, alpha), the rate-limiting enzyme of phosphatidylcholine (PtdCho) synthesis, during autophagy. We also discovered that the loss of PCYT1A activity results in compromised autophagosome formation and maintenance in autophagic cells. Direct tracing of ChoPLs with fluorescence and immunogold labeling imaging revealed the incorporation of newly synthesized ChoPLs into autophagosomal membranes, endoplasmic reticulum (ER) and mitochondria during anticancer drug-induced autophagy. Significant increase in the colocalization of fluorescence signals from the newly synthesized ChoPLs and mCherry-MAP1LC3/LC3 (microtubule-associated protein 1 light chain 3) was also found on autophagosomes accumulating in cells treated with autophagy-modulating compounds. Interestingly, cells undergoing active autophagy had an altered ChoPL profile, with longer and more unsaturated fatty acid/alcohol chains detected. Our data suggest that *de novo* synthesis may be required to increase autophagosomal ChoPL content and alter its composition, together with replacing phospholipids consumed from other organelles during autophagosome formation and turnover. This addiction to *de novo* ChoPL synthesis and the critical role of PCYT1A may lead to development of agents targeting autophagy-induced drug resistance. In addition, fluorescence imaging of choline phospholipids could provide a useful way to visualize autophagosomes in cells and tissues.

**Abbreviations:**

AKT: AKT serine/threonine kinase; BAX: BCL2 associated X, apoptosis regulator; BECN1: beclin 1; ChoPL: choline phospholipid; CHKA: choline kinase alpha; CHPT1: choline phosphotransferase 1; CTCF: corrected total cell fluorescence; CTP: cytidine-5ʹ-triphosphate; DCA: dichloroacetate; DMEM: dulbeccos modified Eagles medium; DMSO: dimethyl sulfoxide; EDTA: ethylenediaminetetraacetic acid; ER: endoplasmic reticulum; GDPD5: glycerophosphodiester phosphodiesterase domain containing 5; GFP: green fluorescent protein; GPC: glycerophosphorylcholine; HBSS: hanks balances salt solution; MAP1LC3/LC3: microtubule associated protein 1 light chain 3; LPCAT1: lysophosphatidylcholine acyltransferase 1; LysoPtdCho: lysophosphatidylcholine; MRS: magnetic resonance spectroscopy; MTORC1: mechanistic target of rapamycin kinase complex 1; PCho: phosphocholine; PCYT: choline phosphate cytidylyltransferase; PLA2: phospholipase A2; PLB: phospholipase B; PLC: phospholipase C; PLD: phospholipase D; PCYT1A: phosphate cytidylyltransferase 1, choline, alpha; PI3K: phosphoinositide-3-kinase; pMAFs: pancreatic mouse adult fibroblasts; PNPLA6: patatin like phospholipase domain containing 6; Pro-Cho: propargylcholine; Pro-ChoPLs: propargylcholine phospholipids; PtdCho: phosphatidylcholine; PtdEth: phosphatidylethanolamine; PtdIns3P: phosphatidylinositol-3-phosphate; RPS6: ribosomal protein S6; SCD: stearoyl-CoA desaturase; SEM: standard error of the mean; SM: sphingomyelin; SMPD1/SMase: sphingomyelin phosphodiesterase 1, acid lysosomal; SGMS: sphingomyelin synthase; WT: wild-type

## Introduction

Cytoprotective macroautophagy, henceforth referred to as autophagy, is implicated in resistance to a wide range of anticancer treatments by means of mitigating cellular stress [1,2] inflicted by therapy-induced nutrient or growth factor deprivation, energy depletion, hypoxia, accumulation of protein aggregates, damage to organelles or DNA and reactive oxygen species production [3]. It is a highly regulated catabolic process, whereby parts of the cytosol and cellular organelles, including protein aggregates, damaged or superfluous organelles or pathogens, are sequestered into the double-membrane vesicles, called autophagosomes, and delivered to lysosomes for degradation. The resulting “nutrients” are released into the cytosol to support energy metabolism, biosynthesis and cellular homeostasis. Here we investigate the metabolic impact of anti-cancer treatment, starvation and Tat-Beclin 1 induced autophagy in cancer cells.

Autophagy is initiated by the ULK1/2 (unc-51 like autophagy activating kinase 1/2) signaling as well as the activation of the PIK3C3/Vps34 (phosphatidylinositol 3-kinase catalytic subunit type 3) complex, containing BECN1/Beclin-1, which assembles at the phagophore nucleation site to produce phosphatidylinositol-3-phosphate (PtdIns3P) [4]. For example, PtdIns3P contributes to the formation of an ER-associated cup-shaped membrane structure, the omegasome, which acts as a platform for the orchestrated recruitment of other autophagy proteins [5]. This leads to the phagophore expansion at least in part due to the lipidation, and hence membrane association, of the autophagosome marker LC3. The current consensus is that the autophagosome assembly takes place at the ER or, more specifically, ER-mitochondrial junctions during starvation-induced autophagy [5–10]. Until the phagophore detaches from the ER, the phospholipids needed for its expansion are thought to be synthesized locally at the omegasome [11]. It is thought that subsequent autophagosome expansion is driven by vesicles derived from the Golgi and plasma membrane via recycling endosomes [11–13]. In the final stage the phagophore seals to form the autophagosome, which then fuses with the lysosome.

While the source of the autophagosomal membrane during starvation-induced autophagy has been subject to much recent research, the metabolism of major cellular phospholipids, PtdCho and PtdEth (phosphatidylethanolamine) is not well understood. PtdEth has an established role in autophagy as the lipid anchor for LC3, which is required for the elongation of the phagophore and is thought to positively regulate the autophagic flux [14–16].

**Figure 1.**
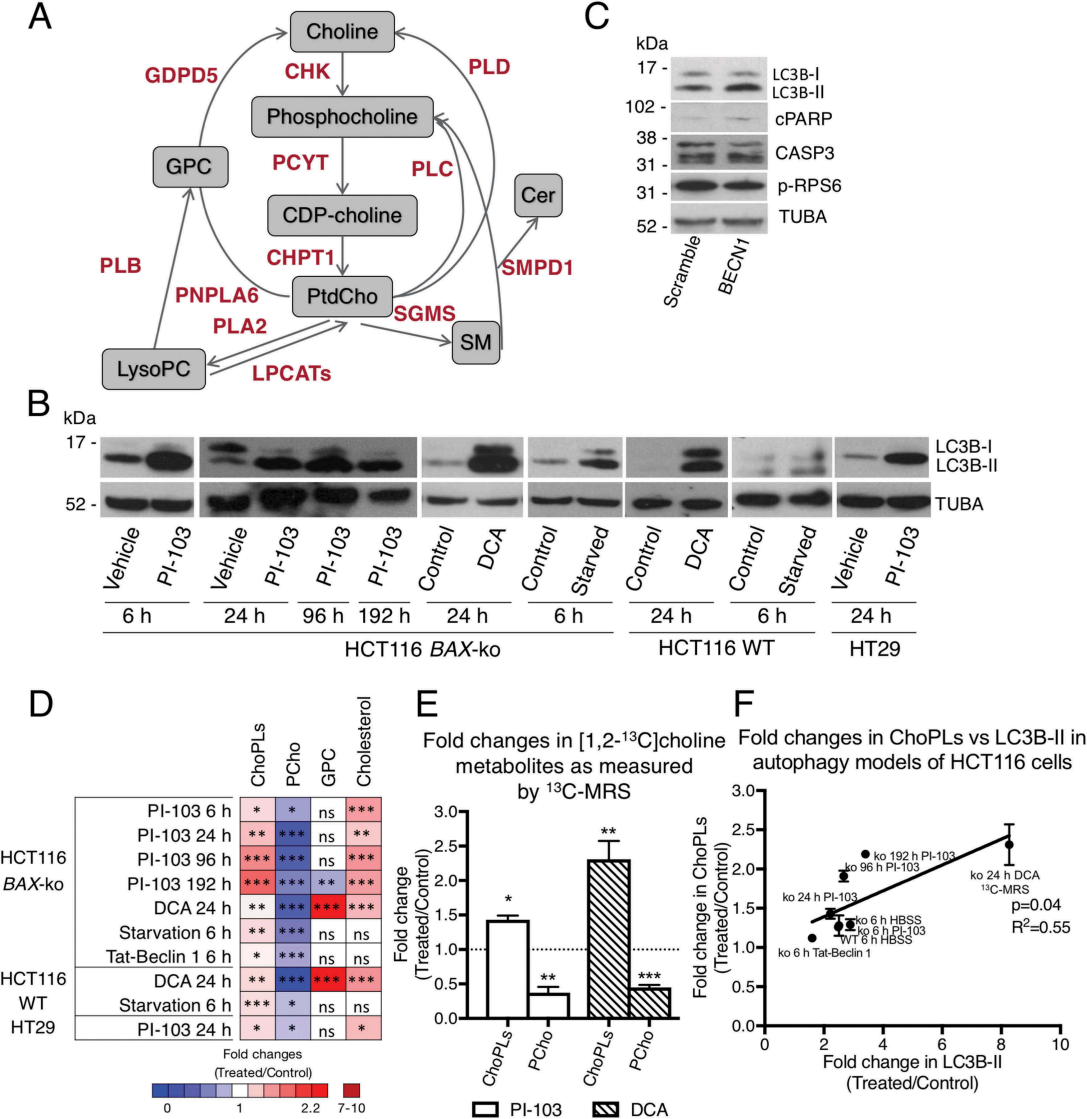
Changes in choline metabolism in cell models of autophagy. (A) A simplified diagram of cellular choline metabolism. Key metabolites are shown in gray boxes, enzymes of choline phospholipid metabolism are shown in red. (B) Western blots of autophagy marker LC3B in 20 μM PI-103 (6, 24, 96 and 192 h) or 75 mM DCA (24 h) or starvation (6 h)-treated HCT116 *BAX*-ko cells, in 75 mM DCA (24 h) and starvation (6 h)-treated HCT116 WT, and in 100 μM PI-103 (24 h)-treated HT29 cells. TUBA was used as a loading control. (C) Western blot of autophagy marker LC3B, apoptosis marker cleaved PARP (cPARP) and CASP3, and p-RPS6 in HCT116 *BAX*-ko cells treated with 50 μM Tat-Beclin 1 or 50 μM Tat-Scramble control for 6 h. TUBA was used as a loading control. (D) Summary of changes in cellular choline metabolites and cholesterol in drug- or starvation-induced autophagy models. Data shown are means of fold changes and presented as a color-coded heat map for different treatment groups compared with their respective controls. (E) Fold changes in [1,2-^13^C]choline metabolites in HCT116 *BAX*-ko cells treated with 20 μM PI-103 (24 h) or 75 mM DCA (24 h). Normal medium was substituted for medium containing [1,2-^13^C]choline instead of unlabeled choline in the last 6 h of treatment. Data expressed as mean ± SEM, n = 3 in each group. (F) ChoPL level as measured by MRS versus LC3B-II expression as measured by western blot densitometry in HCT116 cell autophagy models. Data expressed as fold change (treated/control). Statistically significant changes are indicated: *p < 0.05, **p < 0.01, ***p < 0.001.

The most abundant phospholipids in eukaryotic cell membranes are ChoPLs, which are essential for membrane structure and cellular function. Altered choline metabolism is a metabolic feature of oncogenesis and tumor progression [17]. ChoPLs comprise ester- or ether-linked PtdCho, sphingomyelin (SM) and ester- or ether-linked lysophosphatidylcholines (LysoPtdCho), with PtdChos accounting for more than 50%, SMs contributing a further 5–20%, and LysoPtdChos about 3% of all cellular membrane phospholipids [18,19]. The Kennedy pathway accounts for the bulk of the ChoPL PtdCho biosynthesis (about 80% of all ChoPLs), with PCYT (choline phosphate cytidylyltransferase) being the rate-limiting enzyme and its predominant isoform being encoded by the *PCYT1A* gene (Figure 1A) [20]. Some PtdChos can also be synthesized from LysoPtdCho by Lands cycle enzymes, LPCATs (lysophosphatidylcholine acyltransferases) [21] (Figure 1A). PtdCho can be used for downstream synthesis of LysoPtdCho and SMs.

The relative input of different organelles and the plasma membrane as well as *de novo* synthesis of phospholipids may vary depending on the initiating signal for autophagy and the nutrients available to the cells. Furthermore, replenishing phospholipid sources for autophagic membrane synthesis is required for their continual functioning and for ensuring autophagy-dependent survival during anti-cancer treatment. Consistently, neutral lipid stores were shown to contribute to autophagic membrane phospholipid formation during starvation-induced autophagy [22].

Metabolic stress is a potent physiological stimulus of autophagy, with MTORC1 (mechanistic target of rapamycin kinase complex 1) being a major negative regulator of autophagy [23]. Starvation using a medium lacking amino acids and growth factors has been the system of choice for autophagy studies; however, it may not reflect the actual conditions found in the tumor microenvironment.

In this study, we examined choline phospholipid metabolism in cancer cells during autophagy under both nutrient-poor and nutrient-rich conditions. We used anticancer agents dichloroacetate (DCA), a pyruvate dehydrogenase kinase inhibitor currently in clinical investigation as antineoplastic treatment [24] that we had previously shown to induce autophagy [25], and PI-103, a dual phosphoinositide 3-kinase (PI3K)-AKT (AKT serine/threonine kinase) and MTOR inhibitor that also induces cytoprotective autophagy in drug-resistant glioma and myeloma [26,27], to initiate autophagy in well-nourished conditions. We starved cells in Hanks Balanced Salt Solution (HBSS) to simulate nutrient-poor conditions. We had previously shown that DCA, PI-103 or starvation in HBSS induce autophagy in human colorectal carcinoma wild-type (WT) HCT116, HT29, and the apoptosis-resistant HCT116 *BAX* (BCL2 associated X, apoptosis regulator)-knockout (ko) cells, which is associated with MTORC1 inhibition [25,28,29]. We also treated HCT116 *BAX*-ko cells with Tat-Beclin 1, an autophagy-inducing peptide derived from BECN1, to induce autophagy independently of MTORC1 [30].

With a novel approach to directly image newly synthesized ChoPLs, we showed their incorporation into autophagosomal membranes. We found increased expression of the rate-limiting enzyme of PtdCho synthesis, PCYT1A, in cancer cells undergoing autophagy and showed that the loss of its activity results in the inability of cells to maintain autophagosome biogenesis. Finally, using targeted mass spectrometry, we found that the increased level of choline phospholipids in autophagy was accompanied by an altered profile of choline phospholipid species.

## Results

### Cancer cells undergoing autophagy showed increase in choline phospholipids and decrease in phosphocholine levels

Consistent with our previous findings [25,28], autophagy was induced in HCT116 WT, HCT116 *BAX*-ko and HT29 cells with DCA, PI-103 and starvation in HBSS as indicated by an elevated expression of the autophagy marker LC3B-II (Figure 1B). Increased expression of LC3B-II with minimal apoptosis was also observed in Tat-Beclin 1-treated HCT116 *BAX*-ko cells, with no effect on MTORC1, as indicated by unchanged phosphorylation status of MTORC1 downstream effector RPS6 (ribosomal protein S6) when compared to Tat-Scramble control (Figure 1C).

Steady-state magnetic resonance spectroscopy (^1^H-MRS) measurements (Figure S1A and B) revealed altered levels of intracellular choline metabolites in our autophagy models. The cellular level of phosphocholine (PCho) was reduced in all treatments (Figure 1D and Table S1). glycerophosphorylcholine (GPC) was reduced with 192 h of PI-103 treatment in HCT116 *BAX*-ko cells, increased with 24 h DCA treatment in both HCT116 *BAX*-ko and HCT116 WT cells but unchanged in the remaining autophagy models (Figure 1D and Table S1). Despite the reduced levels of PCho, ChoPLs were increased in all treatment groups (Figure 1D and Table S1). Our data suggested that autophagy in colorectal carcinoma cells was accompanied by alterations in the choline metabolic flux. We also measured the cellular levels of cholesterol, as it accounts for about 30% of total membrane lipids [31]. We observed an increase in total cholesterol in drug-treatment induced autophagy but not in Tat-Beclin 1 treatment or starvation models of autophagy (Figure 1D and Table S1), implying that an increase in ChoPLs might be a more universal marker of autophagy.

### Increased choline phospholipid levels in cancer cells undergoing drug-induced autophagy were due to de novo synthesis

To establish whether the increased level of ChoPLs observed in autophagic cells under nutrient-rich conditions was due to an increase in the *de novo* synthesis, we traced [1,2-^13^C]choline metabolites by ^13^C-MRS (Figure S1C and D). Autophagy was induced in HCT116 *BAX*-ko cells with 24 h PI-103 or DCA treatment, and in the last 6 h of the treatment the medium was substituted for that containing [1,2-^13^C]choline instead of the unlabeled choline. The selection of this timing ensured that *de novo* ChoPL synthesis was measured after the onset of autophagy for both treatments (Figure S2). ^13^C-labeled ChoPLs were increased in PI-103-treated and DCA-treated HCT116 *BAX*-ko cells when compared with controls (Figure 1E). ^13^C-labeled PCho was reduced following both treatments (Figure 1E). No ^13^C-labeled GPC was detected during this time-course. These results indicated that *de novo* ChoPLs synthesis from choline was increased during drug-induced autophagy.

We next sought to understand the relationship between the levels of ChoPLs and LC3B-II since a positive correlation between LC3-II and the number of autophagosomes has been established [14,32]. Linear regression analysis of the fold increase in cellular ChoPLs, as measured by MRS, *versus* the fold increase in LC3B-II, as measured by western blot, showed a significant (p = 0.04) positive (R^2^ = 0.55) relationship between the two (Figure 1F). However, ChoPLs, and specifically the major PtdCho species, are intermediary metabolites involved in other metabolic processes as well. Thus, with DCA-induced autophagy, there was a large increase in the PtdCho breakdown product GPC, which was specific for the DCA treatment, but not in other autophagy models, suggesting that this effect was unrelated to autophagy (Figure 1D). We observed a large increase in LC3B-II in DCA-treated cells, and indeed, our data from ^13^C-choline tracing (Figure 1E) indicated highly elevated *de novo* synthesis of ChoPLs. However, the steady-state level of ChoPLs (Figure 1D) was increased to a much lesser extent, which might be a result of the net change in increased PtdCho synthesis and breakdown of PtdCho to GPC. We therefore used ^13^C-choline tracing data for DCA treatment for Figure 1F.

### Newly synthesized choline phospholipids contributed to the phagophore membrane

**Figure 2.**
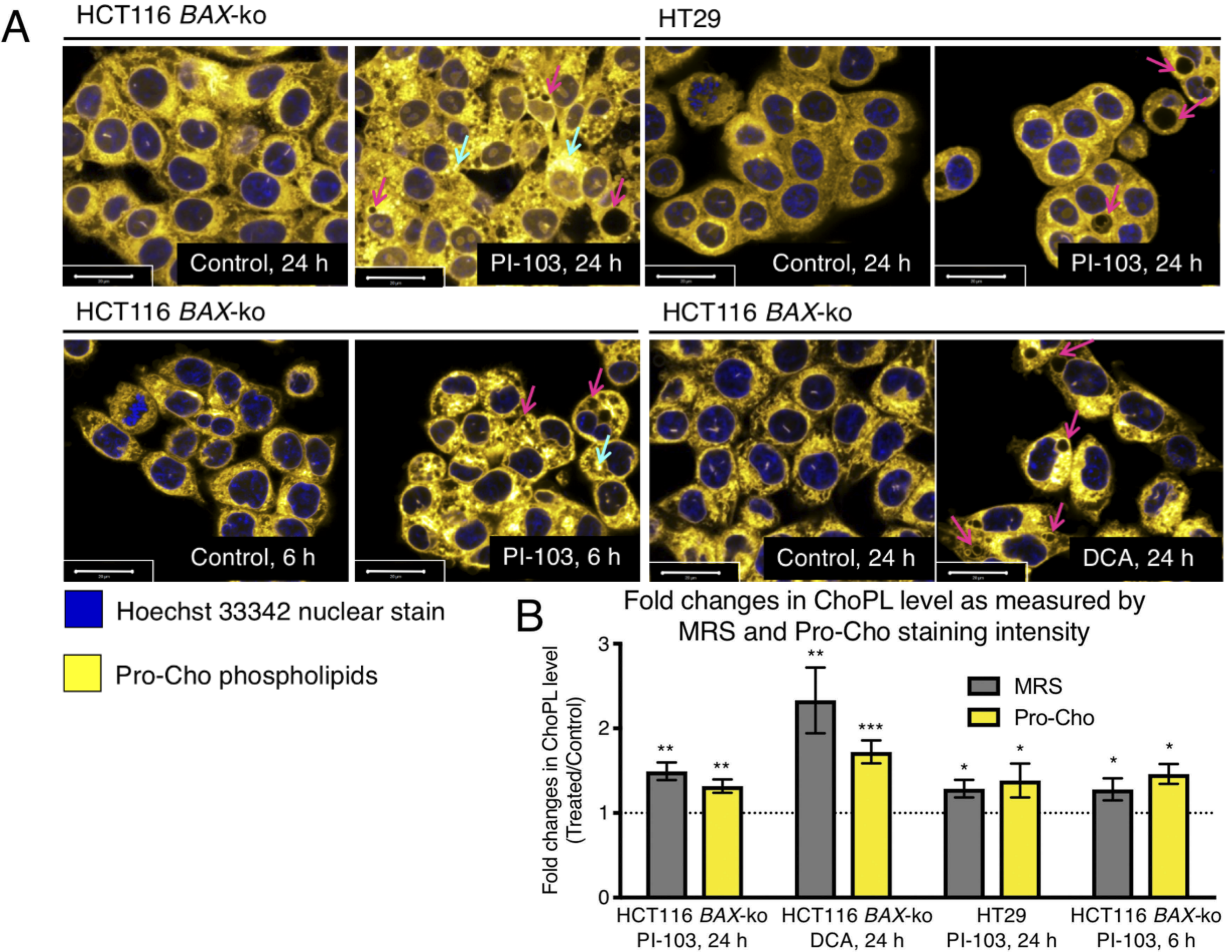
Increased synthesis and vacuolar appearance of Pro-Cho-labeled phospholipids in drug-induced autophagic cells. (A) Imaging of Pro-ChoPLs in 20 μM PI-103 (24 h and 6 h)-treated HCT116 *BAX*-ko cells, 100 μM PI-103 (24 h)-treated HT29 cells and in 75 mM DCA (24 h)-treated HCT116 *BAX*-ko cells. Pro-Cho was added together with the PI-103 treatment but in the last 6 h of DCA treatment to adjust for the difference in the timing of autophagy onset between the two treatments (Figure S2). The cells were then stained with Alexa Fluor 647-azide. Magenta arrows indicate potential autophagosomes or autolysosomes as vesicles enclosed in Pro-ChoPL membranes, cyan arrows indicate potential autophagosomes with autophagic cargo containing Pro-ChoPLs. Scale bar 20 μm. (B) Fold changes in ChoPL level in drug-induced autophagy as measured by ^1^H-MRS (for PI-103 treatments) and ^13^C-MRS (for DCA treatment) in comparison to corrected total cell fluorescence as obtained by propargyl-choline incorporation and staining. Data expressed as mean ± SEM, min *n* = 3 in each group. Statistically significant changes are indicated: *p < 0.05, **p < 0.01, ***p < 0.001.

We next imaged the metabolic fate of the newly synthesized ChoPLs in cells undergoing autophagy to determine if they contribute to the newly synthesized membrane phospholipids in the forming and expanding phagophores, or replenish ChoPLs in other organelles, such as the ER and mitochondria, which supply membranes for the growing phagophores. We used propargylcholine (Pro-Cho), a synthetic analog of choline, which is converted via the Kennedy pathway to propargyl-PtdCho and all other classes of ChoPLs, albeit its incorporation into SM and ether PtdCho is somewhat slower [33]. Pro-Cho addition does not alter the relative abundance of other membrane phospholipids [33]. Treatment media were supplemented with Pro-Cho at the start of the 6 h or 24 h treatment with PI-103 in HCT116 *BAX*-ko and HT29 cells, and in the last 6 h of 24 h DCA treatment of HCT116 *BAX*-ko cells, to account for the temporal differences in autophagy onset between DCA and PI-103 treatments (Figure S2). Fluorescent Pro-ChoPL staining of the nuclear envelope and structures consistent with the ER and mitochondria was visible in both control and in PI-103- or DCA-induced HCT116 *BAX*-ko and HT29 cells undergoing autophagy (Figure 2A). In addition, Pro-ChoPLs also enclosed and localized to vesicular structures that are likely to be autophagic vesicles (Figure 2A).

The corrected total cell fluorescence (CTCF) measurements of Pro-ChoPLs were higher in the drug-induced autophagic cells when compared to their respective controls (Figure 2B). These values were in a good agreement with the fold increases in ChoPLs measured by ^1^H-MRS (for HT29 cells) and ^13^C-MRS (for HCT116 *BAX*-ko cells) (Figure 2B). The fold increases in CTCF are mostly likely reflecting the increases in PtdCho levels [33], as it is the most abundant class of choline phospholipid (about 80% of all ChoPLs) present in mammalian cell membranes.

**Figure 3.**
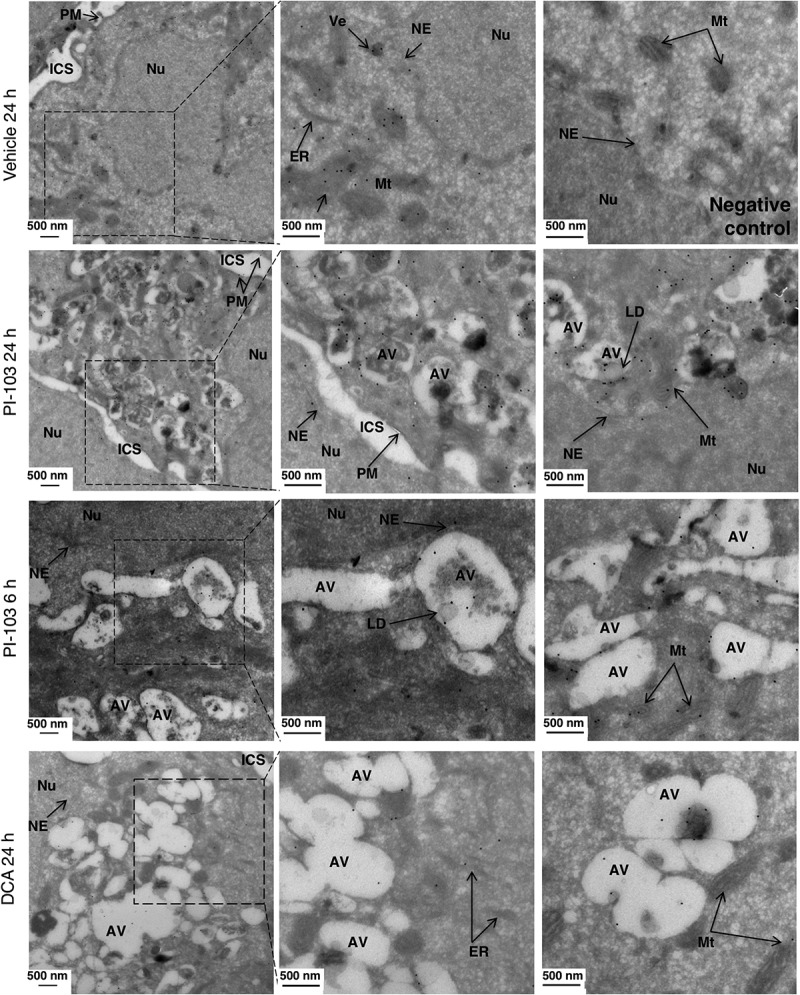
Localization of Pro-ChoPLs during drug-induced autophagy. Imaging of Pro-ChoPLs in 20 μM PI-103 (24 h and 6 h), and in 75 mM DCA (24 h)-treated HCT116 *BAX*-ko cells by immuno-electron microscopy. Pro-Cho was added together with the vehicle- or PI-103 treatment, but in the last 6 h of DCA treatment to adjust for the difference in the timing of autophagy onset between the two treatments (Figure S2). No Pro-Cho was added for the negative, vehicle-treated control. The cells were fixed, sectioned and Pro-ChoPLs reacted with biotin-azide. The sections were incubated with anti-biotin antibodies and protein A gold, counterstained with uranyl acetate and imaged by transmission electron microscopy. Arrows indicate various cellular structures: AV, autophagic vacuole; ER, endoplasmic reticulum; ICS, intercellular space; Mt, mitochondrion; NE, nuclear envelope; Nu, nucleus; PM, plasma membrane; Ve, vesicle.

To determine the precise localization of the newly synthesized ChoPLs in drug-induced autophagy, HCT116 *BAX*-ko cells were labeled with Pro-Cho as above and the position of Pro-ChoPLs was detected using immunogold labeling and imaging by transmission electron microscopy. Gold particles were localized to the nuclear envelope, the ER, mitochondria, plasma membrane and small vesicular structures in control cells, whereas the nucleus and areas of cytosol without membrane structures were largely devoid of gold label (Figure 3). More gold label was observed in both PI-103 and DCA-treated cells when compared to controls and they were visible on the membranes of autophagic vacuoles, as well as in digested material inside the autolysosomes (Figure 3). Furthermore, we also observed electron-dense round structures with gold-labeled Pro-ChoPLs, consistent with lipid droplets, in close proximity to or inside autophagosomes (Figure 3). These may reflect lipophagy and/or the transfer of Pro-ChoPLs between the lipid droplets and the autophagic structures [22,34]. We concluded that the newly synthesized ChoPLs contribute to the phagophore and, subsequently, autolysosomal membranes.

### Fluorescence signals from the newly synthesized choline phospholipids colocalized with mCherry-LC3 in pMAFs (pancreatic mouse adult fibroblasts) undergoing drug-induced autophagy

To further corroborate the colocalization of the newly synthesized ChoPLs with autophagosomes, pMAFs expressing mCherry-GFP-LC3 were labeled with Pro-Cho, and the degree of colocalization of Pro-ChoPLs with LC3 was detected using confocal microscopy. Increased mCherry-LC3 signal was found in pMAFs cells treated with bafilomycin A_1_, Torin-1 or PI-103 when compared with dimethyl sulfoxide (DMSO) control, confirming the induction of autophagy and autophagosome accumulation (Figure S3A). No green fluorescence signal was observed for GFP, due to the steps in the click chemistry reaction that involve a very low pH which led to the quenching of GFP signal. Hence, only red florescence signals from mCherry-LC3 were observed (Figure S3A). In order to confirm the specificity of the Pro-Cho fluorescence probe, a negative control was utilized in which click chemistry was performed on pMAFs cells using the Alexa Fluor 350-azide probe without prior incubation with Pro-Cho. No signal from the Alexa Fluor 350-azide probe was observed (Figure S3A). This experiment confirmed the specificity of the Alexa Fluor 350 probe and the absence of its interference with the mCherry signal.

**Figure 4.**
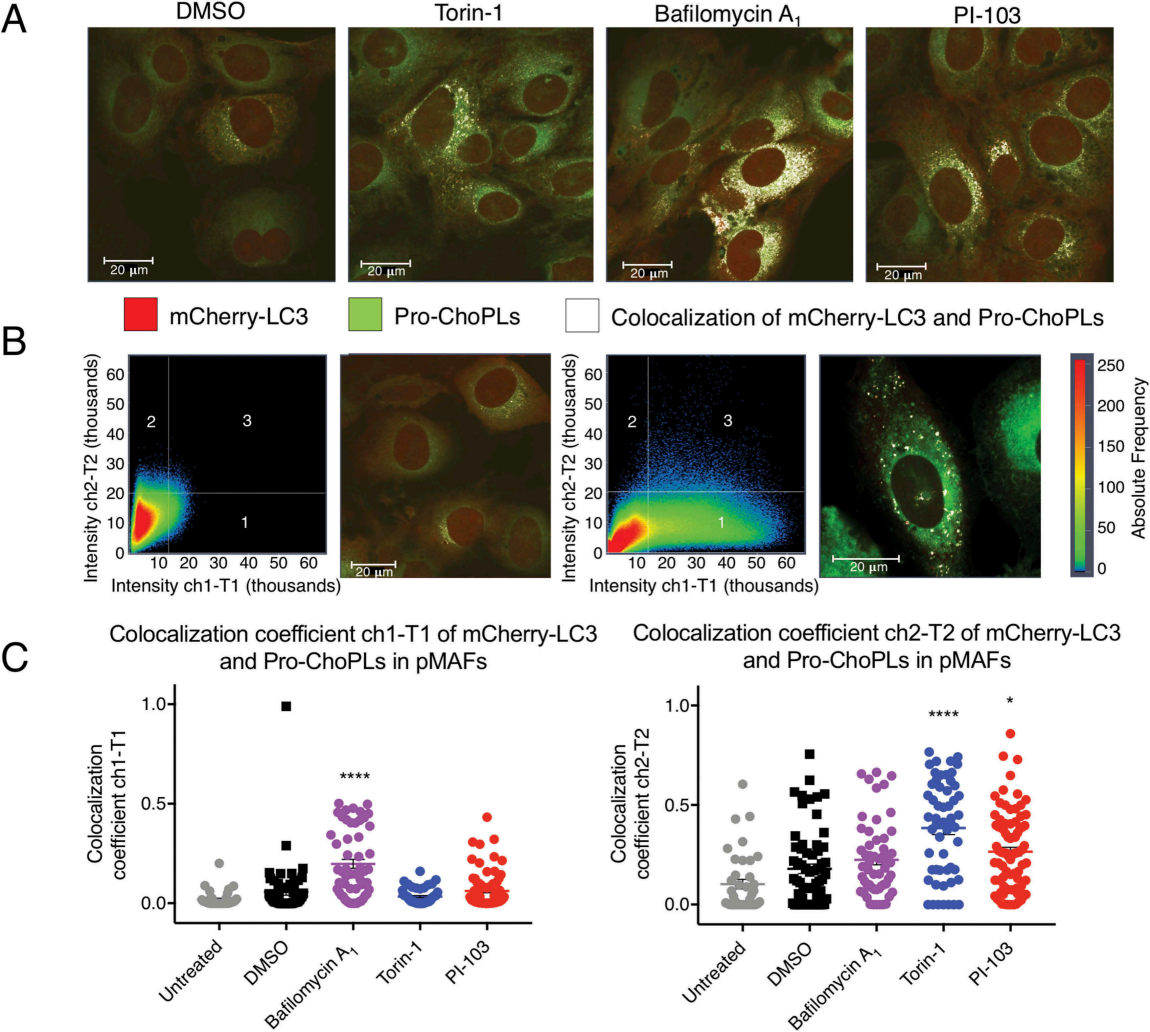
Colocalization of Pro-ChoPLs and mCherry-LC3 fluorescence signals in drug-induced autophagic mCherry-GFP-LC3 pMAFs. (A) Imaging of Pro-ChoPLs and mCherry-LC3 in mCherry-GFP-LC3 pMAF cells following 6 h of 250 nM Torin-1, 200 nM bafilomycin A_1_, 20 µM PI-103 or DMSO treatment. These pMAF cells were treated in the last 6 h before the end of 24 h incubation with Pro-Cho and then stained with Alexa Fluor 350-azide. The Pro-ChoPL signals were pseudo-colored to green using the Zeiss software on the microscope, in order to enhance the contrast against the red signal. The yellow signals (colocalization of the Pro-ChoPLs and mCherry-LC3 staining) were artificially colored to white using the software on the microscope, in order to enhance the contrast against the red and green signals. Scale bar: 20 μm. (B) Colocalization Pro-ChoPLs and mCherry-LC3 showed in a scatterplot (pixels in quadrant 3) and fluorescence image (white signals) in DMSO- or bafilomycin A_1_-treated mCherry-GFP-LC3 pMAFs. (C) The colocalization coefficient ch1-T1 and colocalization coefficient ch2-T2 in mCherry-GFP-LC3 pMAF cells following various treatments. Bars represent mean ± SEM. ****P < 0.0001; *P < 0.05 when compared to DMSO controls.

Pro-ChoPL signal intensities were found to increase in cells treated with bafilomycin A_1_, Torin-1 or PI-103 when compared to the DMSO-treated control (Figure S3B). The labeled Pro-ChoPLs (blue) signals were pseudo-colored to green using the Zeiss software on the microscope, in order to enhance the contrast against the red signal (Figure. 4A and S3B). Significant increase in the colocalization of Pro-ChoPLs and mCherry-LC3 signals was also found in treated cells when compared with DMSO control (Figure 4 and S3B), confirming the colocalization of the Pro-ChoPLs and the mCherry-LC3 signals upon treatment with autophagy-modulating drugs. We concluded that the colocalization of the signals from the newly synthesized Pro-ChoPLs and mCherry-LC3 increased significantly following autophagy induction and the autophagosome accumulation.

### Increased expression of the active membrane-bound form of PCYT1A was associated with increased ChoPLs synthesis in autophagic cells

**Figure 5.**
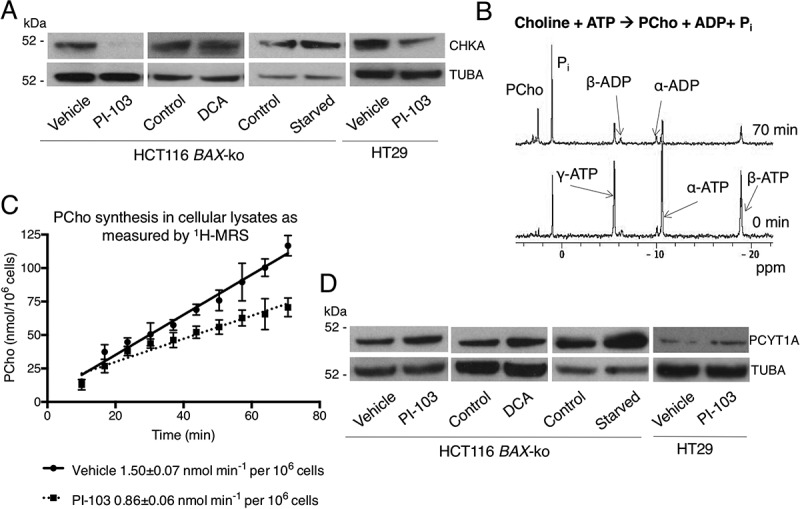
Changes in CHKA and PCYT1A expression and activation in autophagy models. (A) Western blots of CHKA, the main CHK isoform, in 20 μM PI-103 (24 h)-, 75 mM DCA (24 h)-treated and in 6 h starved (in HBSS) HCT116 *BAX*-ko cells and in 100 μM PI-103 (24 h)-treated HT29 cells, TUBA was used as a loading control. (B) Choline kinase activity measurements: ^31^P NMR of the extracted cytoplasm of vehicle (24 h)-treated HCT116 *BAX*-ko cells after addition of exogenous choline, ATP and MgCl_2_ at the start and at the end of the measurement. (C) The increase of PCho peak integral over time in the soluble HCT116 *BAX*-ko cell lysates after the addition of exogenous choline, ATP, and MgCl_2_. The cells were lysed after 24 h of treatment with DMSO control or 20 μM PI-103. A linear fit to the data yields the rate constant. Data expressed as mean ± SEM, *n = 3*. (D) Western blots of PCYT1A in HCT116 *BAX*-ko cells following 20 μM PI-103 (24 h), 75mM DCA (24 h) or HBSS (6 h; starved) treatment, and in HT29 cells following 100 μM PI-103 (24 h) treatment. TUBA was used as loading control.

To determine the mechanisms responsible for the observed reduction in PCho and increase in PtdCho during autophagy, we examined the expression level of the main isoform of choline kinase – CHKA (choline kinase alpha), the first enzyme in the Kennedy pathway of PtdCho synthesis. The expression of CHKA was reduced in both HCT116 *BAX*-ko and HT29 cells treated with PI-103 for 24 h, but unchanged in 24 h DCA-treated and 6 h starved HCT116 *BAX*-ko cells (Figure 5A). These findings were consistent with previous reports of reduced PCho levels and reduced choline kinase expression in PI-103-treated cancer cells [35,36].

To examine how the reduction of CHKA protein level with PI-103 treatment affects the rate of PCho synthesis, choline kinase activity was assayed in HCT116 *BAX*-ko cell lysates using ^31^P-MRS after the addition of exogenous choline, ATP and MgCl_2_ (Figure 5B). Choline kinase activity was lower (58 ± 6%, p = 0.002) in lysates of HCT116 *BAX*-ko cells treated with PI-103 for 24 h (0.86 ± 0.06 nmol min^−1^ per 10^6^ cells) when compared with vehicle-treated controls (1.50 ± 0.07 nmol min^−1^ per 10^6^ cells) (Figure 5C), which was consistent with the decreased PCho level in PI-103-treated cells (Figure 1D). Increased *de novo* ChoPL synthesis was found in cells with active autophagy, despite decreases in PCho and CHKA expression and activity. Hence, our data suggested that the observed increase of ChoPLs in cells undergoing drug-induced autophagy was not dependent on CHKA expression or activity, consistent with it not being rate-limiting in the biosynthesis of PtdCho, the most abundant ChoPL species [37].

The rate-limiting enzyme in the PtdCho biosynthesis pathway, PCYT, catalyzes the production of CDP-choline from cytidine-5ʹ-triphosphate (CTP) and PCho [37]. We saw increased expression of the main isoform of PCYT, PCYT1A, in all our treatment groups when compared to controls (Figure 5D and S4A). The main isoform, PCYT1A, becomes activated when the inactive cytosolic form associates with cellular membranes [38]. When the membrane fraction of PCYT1A was isolated from the cytosolic fraction using digitonin permeabilization [39], an increase in the expression of the active, membrane-bound form of PCYT1A was seen in all of our autophagy models (Figure S4B). These data indicate that higher levels of PCYT1A in its activated form are expressed in autophagic cells.

### Loss of PCYT1A activity abrogated autophagy

**Figure 6.**
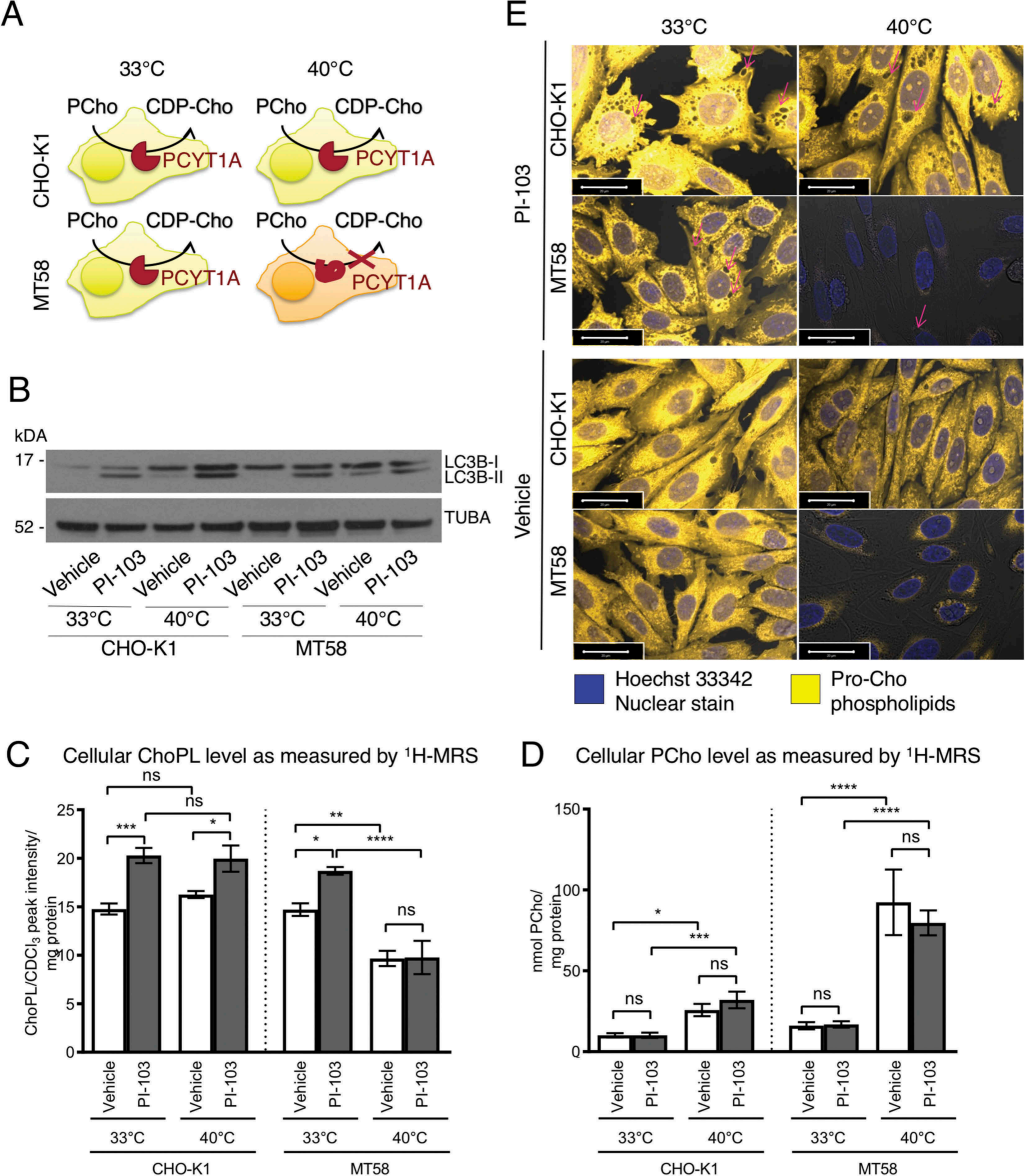
PI-103-induced autophagy and ChoPLs in cells with impaired PCYT1A activity. (A) Schematic of PCYT1A activity in wild type Cho cell line CHO-K1 and in MT58 cells, that contain a temperature sensitive mutation in PCYT1A, at the permissive temperature of 33°C and the restrictive temperature of 40°C. (B) Western blots of LC3B autophagy marker in DMSO (24 h)- and in 11.4 μM PI-103 (24 h)-treated CHO-K1 and MT58 cells at 33°C and 40°C. TUBA was used as a loading control. ChoPL (C) and PCho (D) levels in DMSO (24 h)- and PI-103 (24 h)-treated CHO-K1 and MT58 cells as measured by ^1^H-MRS. Data expressed as mean ± SEM, *n* = 3 in each group. Statistically significant changes are indicated: *p < 0.05, **p < 0.01, ***p < 0.001, ****p < 0.0001. (E) Imaging of Pro-ChoPLs in DMSO (24 h)- and PI-103 (24 h)-treated CHO-K1 and MT58 cells at 33°C and 40°C. Pro-Cho was added together with the PI-103 treatment and the cells were then stained with Alexa Fluor 647-azide. Magenta arrows indicate potential autophagosomes or autolysosomes as vesicles enclosed in Pro-ChoPL membranes. Scale bar 20 μm.

To study the cellular effect of ChoPLs and PCYT1A activity on the autophagy process, we used the Chinese hamster ovary (Cho)-derived MT58 cell line, which contains a temperature-sensitive inactivating mutation in PCYT1A [40]. The wild-type CHO-K1 cells have the same PCYT1A activity at both 33°C and 40°C, whereas raising the temperature from 33°C to 40°C causes the loss of PCYT1A protein stability and function in CHO MT58 cells, allowing us to examine the effect of acute loss of PCYT1A on the autophagy process (Figure 6A). There was no difference in the 4 d GI_50_ for PI-103 between the two cell lines (MT58: 270 ± 30 nM; CHO-K1: 290 ± 20 nM). MT58 and wild-type CHO-K1 cells were treated for 24 h with 11.4 μM PI-103 to achieve ca. 70% reduction in cell number when compared to control cells at 33°C, and ca. 50% reduction at 40°C. The increased level of LC3B-II confirmed the induction of autophagy in both cell lines with PI-103 treatment at both 33°C and 40°C (Figure 6B). The elevated autophagy marker LC3B-II in vehicle-treated MT58 cells at 40°C is likely to be a stress response to the loss of PCYT1A activity (Figure 6B). Unlike the CHO-K1 cells, MT58 cells did not have increased LC3B-II levels with PI-103 treatment at 40°C when compared to 33°C (Figure 6B).

Both CHO-K1 and MT58 cells had an increased level of ChoPLs after the 24 h treatment with PI-103 compared to control at the permissive temperature of 33°C (Figure 6C). However, at the restrictive temperature of 40°C, the vehicle-treated MT58 cells had a reduced level of ChoPLs that was not increased upon PI-103 treatment (Figure 6C). Consistent with the inhibition of PCYT1A activity at 40°C, vehicle-treated MT58 cells at 40°C had a buildup of PCho, the substrate of PCYT1A, in agreement with previous findings [40] (Figure 6D).

Pro-Cho labeling indicated an increase in Pro-ChoPLs in PI-103-treated CHO-K1 cells at both temperatures when compared to vehicle-treated controls, whereas an increase in Pro-ChoPLs was only seen at 33°C in PI-103-treated MT58 cells, consistent with the ^1^H-MRS data (Figure 6E). The amount of Pro-ChoPLs was severely reduced in MT58 cells, indicating a large reduction in new ChoPL synthesis at the restrictive temperature of 40°C (Figure 6E). Very few autophagosomes could be seen by Pro-Cho labeling in PI-103-treated MT58 cells (Figure 6E). A small reduction in the level of Pro-Cho-derived ChoPLs was also seen at 40°C in PI-103- and vehicle-treated CHO-K1 cells and this might have been due to the heat effects. Light microscopy indicated the presence of vacuoles following PI-103 treatment in both cell lines, but fewer vacuoles were visible at 40°C in 18 h and 24 h PI-103-treated MT58 cells when the PCYT1A activity was impaired (Figure S5).

**Figure 7.**
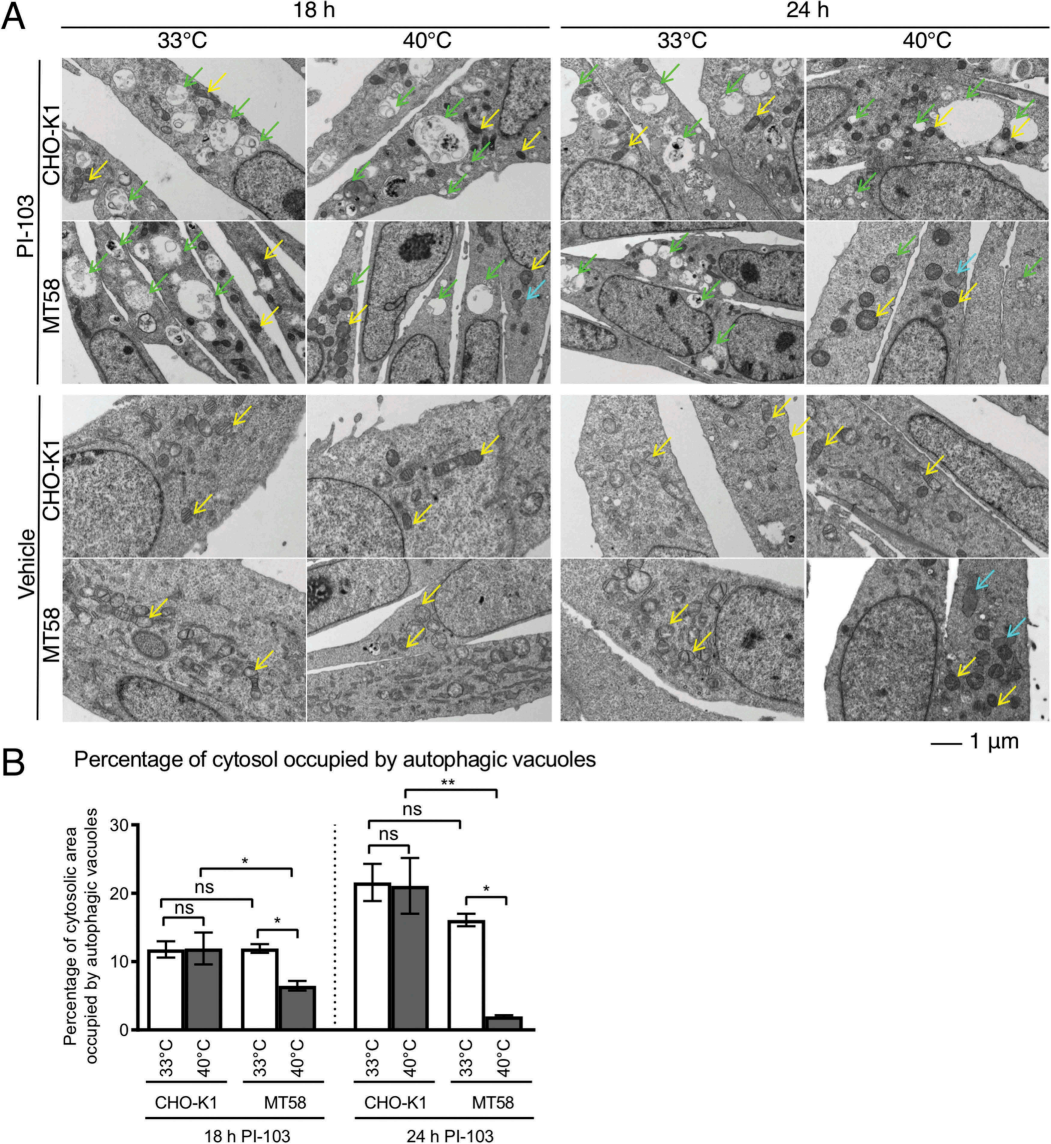
Autophagosome morphology in PI-103-treated cells with impaired PCYT1A activity. (A) Transmission electron micrographs of 18 h- and 24 h- DMSO- and 11.4 μM PI-103-treated CHO-K1 and MT58 cells at the permissive temperature of 33°C and the restrictive temperature of 40°C. Green arrows indicate autophagosome and autolysosomes, yellow arrows indicate mitochondria and cyan arrows indicate ER sphericles. (B) The percentage of cytosolic area occupied by autophagic vacuoles in the transmission electron micrographs shown in (A). Data expressed as mean ± SEM, min *n* = 3 in each group. Statistically significant changes are indicated: *p < 0.05, **p < 0.01.

Electron microscopy was performed on CHO-K1 and MT58 cells following 18 h and 24 h of vehicle and PI-103 treatment at 33°C and 40°C to investigate the dynamics of autophagosome formation. Following 18 h of PI-103 treatment, autophagic vacuoles were present in both CHOK-1 and MT58 cells at 33°C and 40°C, indicating that autophagy could be initiated even when PtdCho synthesis by PCYT1A was impaired (Figure 7A). However, the proportion of cytosolic area occupied by autophagic vacuoles was reduced in MT58 cells at the restrictive temperature at this time-point (Figure 7A,B). After 24 h of PI-103 treatment, the area of cytosol occupied by autophagic vacuoles increased in all PI-103-treated samples, with the exception of the PI-103-treated MT58 cells at 40°C, which had very few autophagic vacuoles present (Figure 7A,B). We concluded that the loss of PCYT1A activity resulted in the inability of cells to sustain autophagosome formation during prolonged periods of autophagy. Consistent with this, even though the levels of LC3B-II were slightly increased in vehicle-treated MT58 cells at 40°C, there were no autophagosomes or autophagic vacuoles visible under this condition (Figure 7A).

The loss of PCYT1A activity also resulted in the dilation of the ER and appearance of spherical ER structures in both vehicle- and PI-103-treated MT58 cells at 40°C, consistent with a previous report [41]. We observed that the loss of PCYT1A activity also caused alterations in mitochondrial morphology, with the shape of mitochondria becoming more rounded, with no apparent deformations of mitochondrial cristae (Figure 7A). Thus, the impairments of PtdCho synthesis resulted in disruption of normal ER and mitochondrial appearance.

### Phospholipid architecture was altered in autophagy

**Figure 8.**
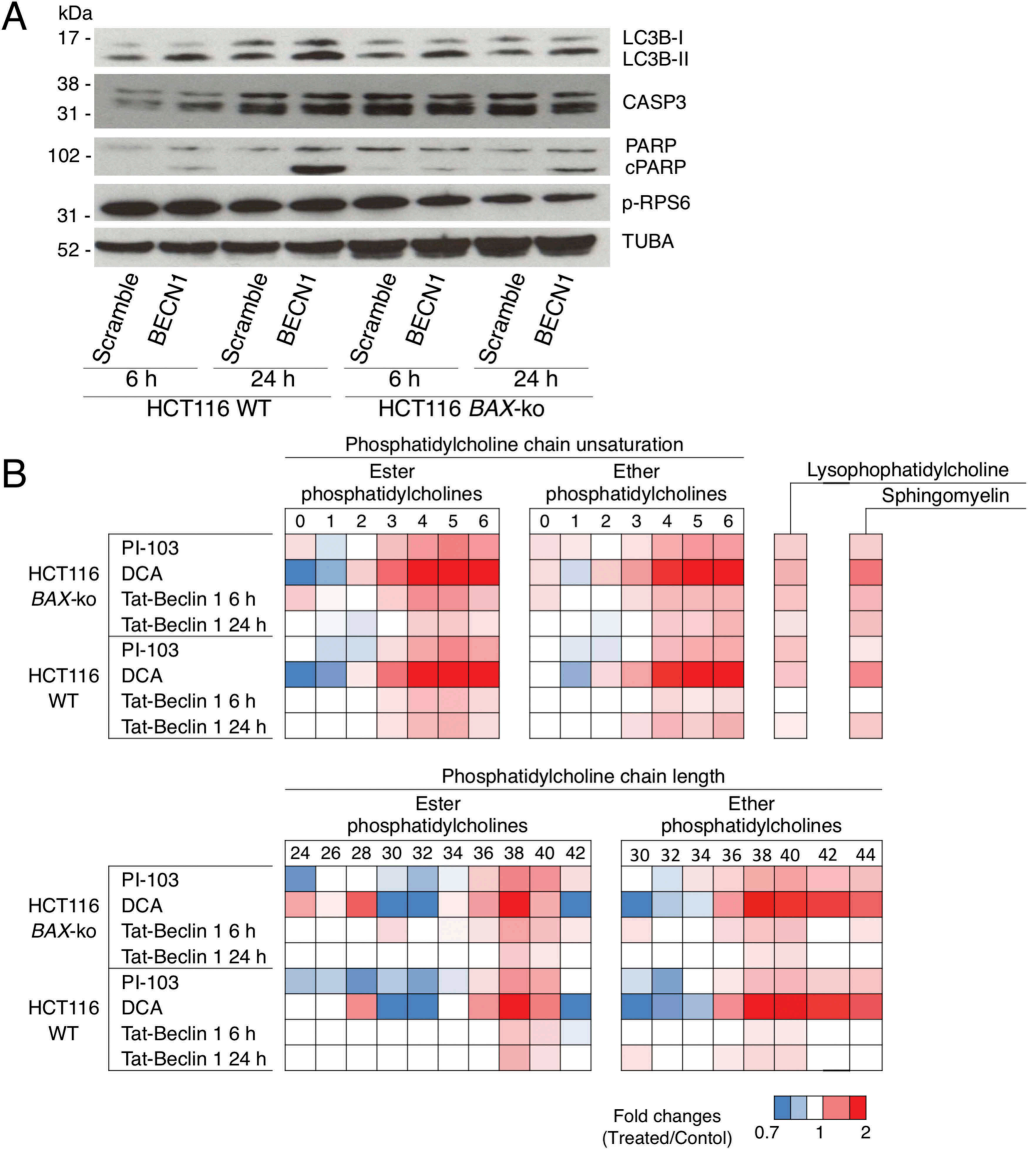
Choline phospholipid composition of autophagic cells. (A) Western blot of autophagy marker LC3B, apoptosis marker cleaved PARP (cPARP) and CASP3, and p-RPS6 in 6 h- or 24 h- HCT116 WT and HCT116 *BAX*-ko cells treated with 50 μM Tat-Beclin 1 or 50 μM Tat-Scramble control. TUBA was used as a loading control. (B) Fold changes in the composition of the measured PtdChos (ester and ether) in HCT116 *BAX*-ko and HCT116 WT cells treated for 24 h with 20 μM PI-103 or 75 mM DCA, 6 h and 24 h 50 μM Tat-Beclin 1 when compared to their respective controls (DMSO for PI-103, water for DCA and 50 μM Tat-Scramble for 50 μM Tat-Beclin 1). The PtdChos are categorized by the total number of double bonds in the fatty acid/alcohol chains of the phospholipid or the fatty acid/alcohol chain length. Fold changes in the sum of all measured SMs and LysoPtdChos of the same treatments are also indicated. Data expressed as a color-coded heat map of fold changes, *n* = 4 in each group. Only statistically significant changes (p < 0.05) are indicated in colors.

To further characterize the PtdCho and related species that were increased during autophagy, we performed targeted metabolomic analysis using the Absolute IDQ p180 kit to measure the levels of 101 choline-containing phospholipid species in the four choline phospholipid groups: PtdCho ester phospholipids (35), PtdCho ether phospholipids (38), LysoPtdChos (14) and SMs (14). To ensure that the observed changes in phospholipid species were related to autophagy rather than to the more general drug effects, Tat-Beclin 1 peptide-induced autophagy models were added to the analysis (Figure 8A).

In all the autophagy models, for both HCT116 WT and HCT116 *BAX*-ko cell lines, there were increased levels of both ester PtdChos and ether PtdChos (plasmalogens) with longer fatty acid/alcohol chain length (with a total of 36–40 carbon atoms per PtdCho molecule) and higher chain unsaturation (3–6 double bonds per PtdCho molecule) (Figure 8B). The total of all LysoPtdChos and SMs measured also increased in all treatment-induced and Tat-Beclin 1-induced autophagic cells, indicating the involvement of additional pathways of choline phospholipid metabolism. These data suggested that the *de novo* PtdCho synthesis might be important not only to increase the absolute amounts of PtdCho but also to alter the ChoPL profile for the autophagosome and autolysosome membrane formation.

## Discussion

Increased ChoPL synthesis in drug-induced autophagy provided membrane phospholipids for the growing autophagosomes and replaced phospholipids consumed from other organelles during autophagosome formation and degradation. Autophagy induced in colorectal cancer cells in response to starvation, the dual PI3K-MTOR inhibitor PI-103, the metabolic drug DCA, or the autophagy-inducing peptide Tat-Beclin 1, was associated with increased levels of ChoPLs and a reduction in PCho as measured by ^1^H-MRS. Supplying the autophagic cells with exogenous ^13^C-labeled choline resulted in increased label incorporation into ChoPLs, showing that ChoPLs were synthesized *de novo*. Pro-Cho labeling indicated that increased ChoPL synthesis in drug-induced autophagy provided membrane phospholipids for autophagosomal membranes and replaced phospholipids consumed from other organelles during autophagosome formation and turnover, as Pro-Cho labeling of mitochondria and the ER was also observed. This study also demonstrated that the fluorescence signals from the newly synthesized Pro-ChoPLs colocalized with mCherry-LC3 signals following autophagy induction and the colocalization was related to autophagosome accumulation.

The expression level of PCYT1A, the rate-limiting enzyme of PtdCho synthesis, increased in its active, membrane-bound form in autophagy models. Loss of PCYT1A function and subsequently reduced ChoPL synthesis capacity resulted in the CHO MT58 cells’ (harboring a temperature-sensitive mutation in PCYT1A) inability to maintain autophagosome formation.

These findings are in keeping with the work of Dupont *et al*., who showed that lipid droplets are mobilized for use in autophagosome membrane synthesis during starvation, which may avoid the use of preformed membranes for autophagy [22]. In addition, they showed that knockdown of CHPT1 (choline phosphotransferase 1), the last enzyme of the Kennedy pathway of PtdCho synthesis, and of LPCAT2, the Lands cycle enzyme, results in a reduced number of autophagy puncta and LC3 lipidation in starved HeLa cells. While Dupont *et al*. focused on the source of the PtdCho fatty acyl chains in starvation, our study examined the autophagosome membrane genesis from the ChoPL synthesis aspect and identified the requirement of PCYT1A for autophagosome membrane formation and maintenance. While we showed upregulation of PCYT1A expression consistent with increased ChoPL levels in autophagy, the regulation of PCYT1A expression and the signals that lead to its membrane localization during autophagy merits further investigation. A recent study has demonstrated that PCYT1A responds to membrane packing defects induced by conical lipids such as PtdEth, which create a physical torque that promotes the association of PCYT1A to the membrane, activating it to synthesize PtdCho and alleviate the membrane torque [42,43]. Given the known role of PtdEth in initiation of autophagosome formation, sensing of membrane stress in phagophores, as well as in other organelles from which membrane lipids are delivered to the growing phagophore, by PCYT1A, could provide a mechanism for linking PtdCho synthesis to the autophagy process.

We also found increased levels of PtdCho ether and PtdCho ester phospholipids with longer fatty acid/alcohol chain lengths and higher chain unsaturation in cells undergoing autophagy. These changes in PtdCho species profile could modify the physical properties of membranes such as fluidity, thickness and membrane curvature and may be important for the formation of autophagosomes and autolysosomal membranes. For example, the introduction of double bonds and reduced acyl chain length increase the membrane fluidity, whereas increased fatty acyl/alcohol chain length leads to thicker bilayer structures [44]. The right balance of various phospholipid species might ensure suitable physical properties for the membrane dynamics that take place during the autophagy process. Further investigation into the specific species and the remodeling enzymes of choline phospholipids may provide new targets for autophagy inhibition.

Altered PtdCho acyl chain composition in autophagic cells suggested the synthesis of new PtdCho and possibly the remodeling of existing PtdCho species by Landes cycle enzymes as the level of LysoPtdCho was increased. This increase in LysoPtdCho could also potentially be due to PtdCho catabolism in autophagosomes during the autophagy process [45]. The increased level of PtdCho with polyunsaturated fatty acyl chains was consistent with the early electron microscopy studies that noted high electron density in autophagosome membranes, a marker of membrane phospholipid unsaturation [46]. More recently, Ogasawara *et al*. identified SCD (stearoyl-CoA desaturase), which introduces a double bond in stearoyl-CoA, as necessary for the earliest steps of autophagosome formation in starved mouse fibroblasts and HeLa cells [47]. SCD is also important for cancer cell survival in metabolically compromised environments [48]. Further studies of the fatty acyl chain requirements for the dynamic membrane processes that take place during autophagy and the enzymes involved in membrane lipid remodeling could provide novel targets to interfere with autophagy.

Another choline phospholipid species that was significantly increased was SM. Sphingolipids have signaling roles in autophagy [49], but Yamagata *et al*. provided evidence that autophagosome formation in starved yeast requires inositol phosphorylceramide, a sphingolipid structurally similar to the mammalian SM [50]. Excess SM that occurs in SMPD1 (sphingomyelin phosphodiesterase 1, acid lysosomal)-deficiency (Niemann-Pick diseases) prevents autophagosome closure by defective regulation of ATG9 (autophagy related 9) cycling during autophagosome maturation [51]. The role of SM in mammalian autophagosome formation requires future investigation.

The findings presented here are also congruent with bioenergetic considerations: it has long been established that autophagosomal membranes, unlike those of other organelles, are largely devoid of protein [52]. It is energetically more costly to remove proteins from existing membranes than to synthesize new membranes, supporting the finding that autophagosome membranes are synthesized *de novo* [15]. Our work suggests that PtdCho synthesis machinery participates in the dynamics of autophagosome formation and sustaining autophagy, as well as in supplying the membranes of other intracellular organelles, such as ER and Golgi, that contribute phospholipids to autophagosomal membranes [5,6,8,10–13].

This study documented and visualized the incorporation of newly made ChoPLs into autophagosome membranes in the clinically relevant setting of treatment-induced autophagy, in conditions where nutrients are available. *De novo* choline phospholipid synthesis was required for sustaining drug-induced cytoprotective autophagy and may provide a new therapeutic target, such as PCYT1A or other enzymes, against autophagy-acquired drug resistance. While the Kennedy pathway is required for normal cell function and thus targeting PCYT1A in cancer may lead to unfavorable side effects, two groups of adult patients with a functional mutation in PCYT1A have been identified [42,53]. These individuals have bone deformities and progressive visual impairments, or abnormal liver and adipose fat metabolism. While detrimental, it indicates that PCYT1A inhibition may be tolerated in patients for a short time period. Furthermore, as new membrane synthesis is required for cellular growth and division in cancer, inhibition of PCYT1A may have an anti-tumor effect. In addition, fluorescence imaging of Pro-Cho labeling may offer a useful way to visualize newly formed autophagosome membranes.

## Materials and methods

### Cell culture and treatment

All media and reagents for cell culture were purchased from Life Technologies. HCT116 *BAX*-ko and HCT116 WT cells were a kind gift of Dr Bert Vogelstein, Johns Hopkin’s Medical Center, USA via Dr Paul Clarke (ICR) and were cultivated in Dulbecco’s Modified Eagle Medium (DMEM; Life Technologies, 41,965) with 3.97 mM glutamine, 25 mM D-glucose, without sodium pyruvate (Life Technologies, 41,965) supplemented with non-essential amino acids (Life Technologies, 11,140). HT29 cells (American Type Culture Collection, HTB-38) were cultivated in McCoy’s 5A medium with glutamine and HEPES (Life Technologies, 22,330). CHO-K1 cells and CHO MT58 cells (MT58 cells), containing a temperature-sensitive mutation in PCYT1A originally isolated by Esko and Raetz [40], were both kindly donated by Prof Dennis Vance, University of Alberta, Canada. CHO-K1 and MT58 cells were cultured in Ham’s F12 medium (Life Technologies, 11,765). All culture media were supplemented with 10% heat-inactivated fetal bovine serum (Sigma-Aldrich, F2442), 100 U/ml penicillin and 100 μg/ml streptomycin (Life Technologies, 15,070). Cells were incubated in a humidified atmosphere containing 5% CO_2_. HCT116 *BAX*-ko, HCT116 WT and HT29 cells were grown at 37°C, CHO-K1 and MT58 cells were grown at the permissive temperature of 33°C. HCT116 *BAX*-ko and HCT116 WT cells were treated for 24 h with 20 μM PI-103 (Selleck Chemicals, S1038), 75 mM DCA (Sigma-Aldrich, 347,795) or for 6 h or 24 h with serum- and amino acid-deprived medium, HBSS (Life Technologies, 24,020). HT29 cells were treated with 100 μM PI-103. For prolonged autophagy, HCT116 *BAX*-ko cells were treated with 20 μM PI-103 for 8 d, with change of fresh medium every day. CHO-K1 and MT58 cells were treated for 18 h or 24 h with 11.4 μM PI-103 at 33°C or 40°C. Treatment with autophagy inducing peptide, Tat-Beclin 1 (amino acid sequence YGRKKRRQRRRGGTNVFNATFEIWHDGEFGT), and its scrambled control, Tat-Scramble (amino acid sequence YGRKKRRQRRRGGVGNDFFINHETTGFATEW) (both custom-ordered from GeneCust) was performed as described [30]. Briefly, prior to treatment the peptides were dissolved in H_2_O to achieve a stock concentration of 50 mg/ml and diluted into Opti-MEM Reduced-Serum Medium (Life Technologies, 31,985) acidified with 0.15% 6 M HCl. HCT116 *BAX*-ko and HCT116 WT cells were washed with phosphate-buffered saline (PBS, Life Technologies, 20,012) and treated with 50 μM Tat-Beclin 1 or Tat-Scramble for 6 h or 24 h. Cells were washed with PBS and collected by standard trypsin (Sigma-Aldrich, T3924, for HT116 *BAX*-ko and HCT116 WT cells or Life Technologies, 12,605, for HT29 cells) treatment. Cell number, viability and average diameter were measured using Vi-CELL Cell Viability Analyzer (Beckman Colter, Brea, CA, USA).

### mCherry-GFP-LC3 pancreatic mouse adult fibroblast cells

pMAFs were derived from mCherry-GFP-LC3 homozygous mice (kindly provided by Ian Ganley, The University of Dundee). After aseptic dissection, the pancreas was finely minced and digested in a “liberase blendzyme” (Roche, 05401020001) for 60 min at 37°C. Tissue fragments were then plated onto 10-cm culture dishes and allowed to attach. Fibroblasts were seen to “crawl” out of the tissue for up to 10 d. These fibroblasts were harvested and sub-cultured in RPMI 1640 medium (GlutaMAX supplemented, Life Technologies, 72,400,021) with 10% FCS, 50 µM of 2-mercaptoethanol (Sigma Aldrich, M6250) and 50 µM of asparagine (Sigma Aldrich, A4159). The fibroblasts divide rapidly for a few divisions, then slow down and cell numbers remain constant or decline slightly for up to 6–8 weeks. Following on from this “crisis” the cells once again return to fast replication at which point they are considered immortal and suitable for use in ongoing studies.

### Immunoblotting

Western blotting was performed as described previously [28]. Proteins separated by SDS-PAGE were electrophoretically transferred onto “Immobilon-P” membrane (Millipore) and blocked with 5% w:v non-fat milk. The following primary antibodies were used: cleaved-PARP (9541), CASP3/caspase-3 (9665), LC3B (2775), phospho-RPS6/RPS6 (Ser240/Ser244, 2215), RPS6 (2217), TUBA/α-tubulin (2144), ACTB/β-actin (4967), CTNNB1/β-catenin (9562) (all from Cell Signaling Technology), PCYT1A (Santa Cruz Biotechnology, sc-161,447), CHKA (Sigma, HPA024153), horseradish peroxidase (HRP)-conjugated polyclonal goat anti-rabbit (GE Healthcare, NA934V) or rabbit anti-mouse (Dako, P0260) secondary antibodies were used. The protein-antibody interactions were visualized using SuperSignal West Pico Chemiluminescent Substrate (Thermo Scientific, 34,080) and exposed to KODAK BioMax XAR Film (Sigma-Aldrich, F5763).

Protein densitometry was performed using ImageJ v1.48 (NIH) software. Relative optical density was calculated by dividing the densitometry of protein with its respective loading control from the same blot. The values obtained were used to calculate the treatment:control ratios.

### CHK activity assay

CHK activity was assayed using an MRS-based method as described [54], with modifications. Briefly, about 25 million cells were collected by trypsinization and washed in PBS. The cells were lysed on ice for 10 min in 550 μl cell lysis buffer containing 50 mM 3-(N-morpholino) propanesulfonic acid-potassium hydroxide (MOPS-KOH; pH 7.5; Sigma-Aldrich, M1254), 5.5 mM sodium bisulfite, 5 mM ethylenediaminetetraacetic acid (EDTA), 5 mM ethylene glycol tetraacetic acid (EGTA) and EDTA-free protease inhibitor cocktail (Thermo Scientific, 88,266). The homogenate was passed through a fine-tipped needle (27.5 G) and lysed by sonication for 3 periods of 5 s. The lysate was centrifuged at 4°C for 20 min at 16,000 *g*. An aliquot of the supernatant fraction (500 µl) containing soluble proteins was placed in a 5 mm NMR tube (Wilmad, Z272019). Reaction mixture was added into the NMR tube immediately prior to the start of MRS measurement. The reaction mixture was prepared in 50 mM Tris-HCl buffer (pH 8.0). The final concentrations of reagents were 5 mM choline chloride, 25 mM MgCl_2_ and 10 mM ATP and the reaction was performed at 25°C to prevent the degradation of ATP. The total volume of reaction in the assay was 650 μl. ^31^P NMR spectra were acquired using a 30° proton-decoupled pulse sequence, spectral width of 12,000 Hz, 1.65 s relaxation delay and 128 scans per FID on a Bruker 500 MHz spectrometer (Bruker Biospin, Coventry, UK). Line broadening of 5 Hz was applied before Fourier transformation and the peak areas were integrated. The absolute concentrations of metabolites were determined using the ATP at the start of the experiment as a reference. The CHK activities were calculated from the straight-line fit to plots of PCho *versus* time and normalized to cell number.

### ^1^H-MRS of cell extracts

For cellular metabolite analysis, water-soluble and lipid metabolites were extracted using a dual phase extraction protocol [55]. Freeze-dried water-soluble cell extracts were reconstituted in 650 μl D_2_O and 50 μl of 0.75% sodium TSP (3-trimethylsilyl-2,2,3,3‐tetradeuteropropionate) in D_2_O (Sigma-Aldrich, 151,882 and 293,040) for chemical shift calibration and quantification. An aliquot of the sample (500 µl) was placed in a 5 mm NMR tube and the pH adjusted to pH 7 using 0.1 M KOH. Chloroform phase (containing lipid metabolites) was evaporated, and the lipid metabolites were reconstituted in 450 μl of deuterated chloroform and 150 μl of 0.1% tetramethylsilane (TMS) in deuterated chloroform (both from Sigma-Aldrich, 151,823 and 434,876) as the standard of reference.

### ^13^C-labeled choline tracer studies

HCT116 *BAX*-ko cells were cultured and treated for 18 h with control, DCA or PI-103 as described above. The treatment medium was then removed and substituted for medium containing [1,2-^13^C] choline together with the respective treatment for a further 6 h. The [1,2-^13^C] choline medium was prepared by using DMEM without choline (Life Technologies, custom ordered) supplemented with 0.0286 mM [1,2-^13^C] choline (Cambridge Isotopes, CLM-548-0.1). The overall treatment length was 24 h.

### MRS measurements

All spectra were acquired on a 500 MHz spectrometer (Bruker Biospin, Coventry, UK) at 298 K. ^1^H spectra were acquired with 7,500 Hz spectral width, 32,768 time domain points, relaxation delay of 2.7 s and 128 or 256 scans for lipid or water-soluble cell extracts, respectively. The water resonance from soluble cell extract samples was suppressed by a gated irradiation centered on the water frequency. For ^13^C-MRS 13,000 spectral averages were acquired for with 26,000 Hz spectral width, 32,768 time domain points and 6.5 s relaxation delay. The spectra were phased and manually baseline-corrected using Bruker TopSpin-3.1 (Bruker Biospin) and MestRe-C-4.9.9.6 6 (Mestrelab Research) software packages, respectively. Spectral assignments were based on literature values [55–57]. Metabolite levels were standardized to cell number.

### Targeted metabolomics analysis

Extracted intracellular metabolites were measured using Absolute*IDQ®* p180 targeted metabolomics kit according to manufacturer’s instructions (Biocrates Life Sciences AG, Innsbruck, Austria). The cells were lysed in 1.5 ml ice-cold 85% ethanol, scraped into 2 ml microcentrifuge tubes and subjected to three rounds of sonication followed by rapid freeze-thaw. The lysates were sonicated for 15 s, frozen for 30 s in liquid nitrogen, rapidly thawed in a 98°C water bath and returned to ice. After the third round of freeze-thaw cycle, the cell lysates were centrifuged at 16,000 *g* for 10 min at 4°C, and the supernatants were collected and freeze-dried to avoid hydrolysis of unstable metabolites. The lyophilized metabolites were stored at −80°C until analysis. The freeze-dried powder was then resuspended in 85% ethanol and 15% of 10 mM phosphate buffer, pH 7.4 to achieve cell concentration of 40 million cells/ml. 10 μl of the sample was applied to the Absolute*IDQ®* p180 plate.

### Mass spectrometry data acquisition and processing

For quantification of glycerophospholipids and sphingolipids, metabolite measurements were performed using the validated Absolute*IDQ* p180 targeted metabolomics kit (Biocrates Life Sciences AG). The samples were processed in the 96-well format provided as per the instructions of the manufacturer. The sample preparations were injected directly into a Waters Xevo TQ-S mass spectrometer coupled to an Acquity HPLC system (Waters Corporation, Milford, MA USA) by electrospray ionization and mass spectrometric analyses were carried out in positive ion mode with multiple reaction monitoring. The elution profile was as follows (min/flow-rate in μl/min): 0/30, 1.6/30, 2.4/200, 2.8/200, 3/30. The auto-sampler was kept at 10°C.

Data processing and peak integration was performed using MassLynxTM (Waters Corporation) and MetIDQ™ software (Biocrates Life Sciences AG). Quantification of the metabolites was achieved by calculating the area under the curve and using a one-point internal standard calibration with representative internal standards (one unlabeled LysoPtdCho, two unlabeled PtdChos and one unlabeled SM). Metabolite levels were standardized to cell number. The semi-quantitative concentration of 76 PtdChos, 15 SMs and 14 LysoPtdChos obtained represent total concentrations of possible isobars and structural isomers. For SMs and LysoPtdChos, the sum of all species detected was used for comparisons of treatment and control samples. For PtdChos, the sum of individual species by bond number or total chain length were used in comparisons.

### Propargyl-choline synthesis

Propargyl-choline (Pro-Cho) was synthesized using the method described [33]. Briefly, dimethylethanolamine (3.54 ml, 35.3 mmol; Sigma Aldrich, 38,990) was added to a solution of propargyl bromide (80% in toluene, 3.74 ml, 33.6 mmol; Sigma Aldrich, 81,831) in anhydrous tetrahydrofuran (THF; 10 ml; VWR, 44,608.AE) at 0°C under a nitrogen atmosphere. The solution was allowed to warm to room temperature and was stirred for 18 h, after which time the resultant solid was washed with cold THF (3 x 20 ml) and dried *in vacuo* to afford the title compound as an off-white solid (6.05 g, 87% yield).

The sample was analyzed by ^1^H NMR (in MeOD): 4.87 (1H, s), 4.46 (2H, d, *J* = 2.3 Hz), 4.04 (2H, a-dq, *J* = 2.5, 4.8 Hz), 3.62 (2H, m), 3.56 (1H, t, *J* = 2.5 Hz), 3.29 (6H, s); ^13^C NMR (in MeOD): 81.3 (C, t, *J* = 40 Hz), 70.7 (CH, t, 8 Hz), 65.3 (CH_2_, s), 55.5 (CH_2_, s), 55.0 (CH_2_, s), 50.6 (CH_3_x2, s).

### Pro-Cho labeling of cells and detection by confocal fluorescence microscopy

Pro-Cho labeling was performed as described [33]. In essence, HCT116 *BAX*-ko and HT29 cells were grown in 35 mm glass bottom dishes (MatTek, P35G-0-10-C) and labeled with 1 mM Pro-Cho bromide in complete media. Unlabeled cells (with no Pro-Cho added) were used as controls. The cells were washed with PBS and fixed for 15 min (IC Fixation Buffer, eBioscience, 00-8222-49). The plates were then washed with TBS (50 mM Tris-Cl, 150 mM NaCl, pH 7.5) and reacted with fluorescent azide for 30 min. The fluorescent azide reaction mixture was prepared fresh every time in 100 mM Tris (from 1 M stock, pH 8.5) and contained 0.75 mM CuSO4, 20 μM Alexa Fluor 647-azide (from 20 mM stock in DMSO, Life Technologies, A10277) and 75 mM ascorbic acid. The cells were washed with TBS, 0.5M NaCl, and then with TBS again before counterstaining with Hoechst 33,342 (Enzo Life Sciences, 23,491-52-3). Cell images were acquired on confocal laser scanning microscope Zeiss LSM 700 (Carl Zeiss, Jena, Germany).

### Immuno-gold electron microscopy of Pro-Cho-labeled cells

HCT116 *BAX*-ko cells were treated with 1 mM Pro-Cho and PI-103 or DCA as for confocal microscopy. The cells were washed with PBS, detached from the dish in 0.5 mM EDTA in PBS, pelleted and fixed with 4% formaldehyde (prepared from a 16% stock; TAAB, F017/2), 0.1% glutaraldehyde (prepared from a 50% stock purchased, TAAB, G014) in 100 mM sodium phosphate buffer (pH 7.4). Fixed cell pellets were infiltrated with 2.3 M sucrose in PBS, frozen in liquid nitrogen and sectioned on an ultramicrotome at −120°C. The 80–100 nm cryo-sections were laid on formvar/carbon-coated copper grids (Agar Scientific, AGS138), thawed, washed with TBS and stained with 20 μM biotin-azide (Life Technologies, B10184) for 30 min. The biotin azide reaction mixture was prepared fresh every time in 100 mM Tris (from 1 M stock, pH 8.5) and contained 0.75 mM CuSO_4_, 20 μM biotin-azide (from 20 mM stock in DMSO) and 75 mM ascorbic acid. The cells were washed with TBS, 0.5 M NaCl, and TBS again, then blocked in 40 mg/ml bovine serum albumin (Sigma-Aldrich, A3311) in TBS. Biotin was detected using a rabbit anti-biotin antibody (Rockland Immunochemicals, 600-401-098), followed by protein A-gold (10-nm colloidal gold; Boster Biological Technology, GA1054). The grids were counterstained and embedded by incubation with 3% uranyl acetate (Agar Scientific, AGR1260A) in 2% methyl-cellulose (Sigma, M6385). The cells were imaged on a Tecnai G2 Spirit BioTWIN transmission electron microscope (Philips/FEI, Eindhoven, the Netherlands) equipped with an AMT 2k CCD camera (Advanced Microscopy Techniques, Woburn, MA, USA).

### Pro-Cho labeling of mCherry-GFP-LC3 pMAF cells and detection by confocal fluorescence microscopy

The immortalized mCherry-GFP-LC3 pMAFs were cultured in RPMI 1640 media containing GlutaMAX (Life Technologies, 72,400,021) supplemented with 10% FCS (PAN Biotech UK, P30-3702), 50 μM 2-mercaptoethanol (Sigma Aldrich, M6250) and 50 μM asparagine (Sigma Aldrich, A4159). The pMAFs were then plated onto ibidi 96-well μ-plate (Thistle scientific, 89,646) and incubated for 5 h. Pro-Cho labeling and synthesis was performed as described [33]. The cells were labeled with 1 mM Pro-Cho bromide for 24 h in complete media, washed with TBS, fixed with 4% PFA at room temp for 15 min and reacted with 20 μM Alexa Fluor 350-azide (Click Chemistry, A1267-01). All the solutions for click chemistry were made fresh, added immediately and incubated on a shaker for 30 min at room temp. The pMAFs were then washed with TBS-NaCl (50 mM Tris-Cl, 0.5 M NaCl, pH 7.5) followed by TBS and with Vectashield (Vector Laboratories, H-1000) added at the end. The pMAFs cells were treated with 250 nM Torin-1 (Bio-techne, 4247), 200 nM bafilomycin A_1_ (Tocris, 1334), 20 µM PI-103 or DMSO for 6 h before the end of the 24 h incubation with Pro-Cho. Cell images were acquired on a confocal laser scanning microscope, Zeiss LSM700 (Carl Zeiss, Jena, Germany).

### Quantitative colocalization analysis

Samples were viewed on a Zeiss LSM700 confocal microscope at x63 magnification. Colocalization was measured using the Zen 2009 software (Carl Zeiss). Colocalization was performed on a pixel by pixel basis with every pixel in the image plotted onto a scatter diagram. Segmentation was established by using the software to false color the red and green pixels (as single color staining was not possible), so that the placement of the crosshairs can be estimated. Quadrant 3 on the scatterplot shows the colocalized pixels. The colocalized pixels in the images were false colored to white, so that the location of these pixels could be observed more readily. Once these conditions were established, they were subsequently kept constant throughout the analysis. Region of interest (ROI) were drawn around each cell in each field of view, in order to remove the areas of no signal which can skew the results. Colocalization was quantified using the Manders colocalization coefficient, as this is sensitive to colocalization independent of a linear relationship of signal levels to each other [58,59]. Colocalization coefficient ch1-T1 and colocalization coefficient ch2-T2 were analyzed. The colocalization coefficients describe the contribution of each one from two selected channels to the pixels of interest, i.e, the colocalization coefficient ch1-T1 shows the value of how many red pixels colocalize with the green pixels, whereas, the colocalization coefficient ch2-T2 shows the value of how many green pixels colocalize with the red pixels. The values are between 0 and 1 where 1 corresponds to 100% colocalization, 0.2 to 20% and 0 to no colocalization [60].

### Electron microscopy of CHO-K1 and MT58 cells

For transmission electron microscopy (TEM) analysis, cells were fixed for 4 h with 2.5% (v:v) glutaraldehyde in 0.1 M cacodylate buffer (pH 7.4; TAAB, S006), pelleted by centrifugation and post-fixed in 1% (w:v) osmium tetroxide (prepared from a 4% stock, TAAB, O014) in 0.1 M cacodylate buffer for 1 h at 4°C. The cells were then stained for 1 h with 2% uranyl acetate (Agar Scientific, AGR1260A) in water at 4°C before dehydration through a graded ethanol series. Samples were equilibrated with propylene oxide (Sigma-Aldrich, 110,205) before infiltration with TAAB epoxy resin (TAAB, T028) and polymerized at 70°C for 24 h. Ultrathin sections (50–70 nm) were prepared using a Reichert-Jung Ultracut E ultramicrotome, mounted on 150 mesh copper grids and contrasted using uranyl acetate and lead citrate [61]. Samples were examined on a FEI Tecnai 12 transmission microscope (Philips/FEI, Eindhoven, Netherlands) operated at 120 kV. Images were acquired with an AMT 16000M camera (Advanced Microscopy Techniques, Woburn, CA, USA). For measuring cytosolic area occupied by autophagosomes, total autophagosomal area was divided by total cytosolic area (measured by NIH ImageJ digital image analysis software) in a random selection of EM images (min. n = 3).

### Statistics

All assays were performed at least in triplicate and data presented as mean ± 1 SEM. Student’s two-tailed unpaired t-test was used in all statistical analyses unless otherwise stated, with a p value of ≤ 0.05 considered significant. For statistical tests involving CHO-K1 and MT58 cells, One-way ANOVA with Bonferroni post-comparison test (GraphPad Prism 6, GraphPad Software Incorporated) was used and a p value of ≤ 0.05 considered significant. To correlate ChoPL levels with LC3B-II expression, linear regression analysis was performed in GraphPad Prism 7 (GraphPad Software). The Welch t-test was used to compare the colocalization coefficient changes between DMSO and the various treatment groups (GraphPad Prism 7).

## Supplementary Material

Supplemental Material

## References

[1] Sui X, Chen R, Wang Z, et al. Autophagy and chemotherapy resistance: a promising therapeutic target for cancer treatment. Cell Death Dis. 2013;4:e838. PMID:24113172. 24113172 10.1038/cddis.2013.350 PMC3824660

[2] Chen N, Debnath J. Autophagy and tumorigenesis. FEBS Lett. 2010;584:1427–1435. PMID:20035753. 20035753 10.1016/j.febslet.2009.12.034 PMC2843775

[3] Kroemer G, Mariño G, Levine B. Autophagy and the integrated stress response. Mol Cell. 2010;40:280–293. PMID:20965422. 20965422 10.1016/j.molcel.2010.09.023 PMC3127250

[4] Mizushima N, Yoshimori T, Ohsumi Y. The role of Atg proteins in autophagosome formation. Annu Rev Cell Dev Biol. 2011;27:107–132. PMID:21801009. 21801009 10.1146/annurev-cellbio-092910-154005

[5] Axe EL, Walker SA, Manifava M, et al. Autophagosome formation from membrane compartments enriched in phosphatidylinositol 3-phosphate and dynamically connected to the endoplasmic reticulum. J Cell Biol. 2008;182:685–701. PMID:18725538. 18725538 10.1083/jcb.200803137 PMC2518708

[6] Hayashi-Nishino M, Fujita N, Noda T, et al. A subdomain of the endoplasmic reticulum forms a cradle for autophagosome formation. Nat Cell Biol. 2009;11:1433–1437. PMID:19898463. 19898463 10.1038/ncb1991

[7] Itakura E, Mizushima N. Characterization of autophagosome formation site by a hierarchical analysis of mammalian Atg proteins. Autophagy. 2010;6:764–776. PMID:20639694. 20639694 10.4161/auto.6.6.12709 PMC3321844

[8] Ylä-Anttila P, Vihinen H, Jokitalo E, et al. 3D tomography reveals connections between the phagophore and endoplasmic reticulum. Autophagy. 2009;5:1180–1185. PMID:19855179. 19855179 10.4161/auto.5.8.10274

[9] Hailey DW, Rambold AS, Satpute-Krishnan P, et al. Mitochondria supply membranes for autophagosome biogenesis during starvation. Cell. 2010;141:656–667. PMID:20478256. 20478256 10.1016/j.cell.2010.04.009 PMC3059894

[10] Hamasaki M, Furuta N, Matsuda A, et al. Autophagosomes form at ER-mitochondria contact sites. Nature. 2013;495:389–393. PMID:23455425. 23455425 10.1038/nature11910

[11] Lamb CA, Yoshimori T, Tooze SA. The autophagosome: origins unknown, biogenesis complex. Nat Rev Mol Cell Biol. 2013;14:759–774. PMID:24201109. 24201109 10.1038/nrm3696

[12] Puri C, Renna M, Bento CF, et al. Diverse autophagosome membrane sources coalesce in recycling endosomes. Cell. 2013;154:1285–1299. PMID:24034251. 24034251 10.1016/j.cell.2013.08.044 PMC3791395

[13] Yen W-L, Shintani T, Nair U, et al. The conserved oligomeric Golgi complex is involved in double-membrane vesicle formation during autophagy. J Cell Biol. 2010;188:101–114. PMID:20065092. 20065092 10.1083/jcb.200904075 PMC2812853

[14] Kabeya Y, Mizushima N, Ueno T, et al. LC3, a mammalian homologue of yeast Apg8p, is localized in autophagosome membranes after processing. Embo J. 2000;19:5720–5728. PMID:11060023. 11060023 10.1093/emboj/19.21.5720 PMC305793

[15] Girardi JP, Pereira L, Bakovic M. De novo synthesis of phospholipids is coupled with autophagosome formation. Med Hypotheses. 2011;77:1083–1087. PMID:21963355. 21963355 10.1016/j.mehy.2011.09.008

[16] Rockenfeller P, Koska M, Pietrocola F, et al. Phosphatidylethanolamine positively regulates autophagy and longevity. Cell Death Differ. 2015;22:499–508. PMID:25571976. 25571976 10.1038/cdd.2014.219 PMC4326582

[17] Glunde K, Bhujwalla ZM, Ronen SM. Choline metabolism in malignant transformation. Nat Rev Cancer. 2011;11:835–848. PMID:22089420. 22089420 10.1038/nrc3162 PMC4337883

[18] Munder PG, Modolell M, Andreesen R, et al. Lysophosphatidylcholine (lysolecithin) and its synthetic analogues. Immunemodulating and other biologic effects. Springer Semin Immunopathol. 1979;2:187–203.

[19] Zeisel SH. Choline phospholipids: signal transduction and carcinogenesis. Faseb J. 1993;7:551–557. PMID:8472893. 8472893 10.1096/fasebj.7.6.8472893

[20] Sundler R, Akesson B. Regulation of phospholipid biosynthesis in isolated rat hepatocytes. Effect of different substrates. J Biol Chem. 1975;250:3359–3367. PMID:1123345. 1123345

[21] Shindou H, Hishikawa D, Harayama T, et al. Recent progress on acyl CoA: lysophospholipid acyltransferase research. J Lipid Res. 2009;50(Suppl):S46–51. PMID:18931347. 18931347 10.1194/jlr.R800035-JLR200 PMC2674719

[22] Dupont N, Chauhan S, Arko-Mensah J, et al. Neutral lipid stores and lipase PNPLA5 contribute to autophagosome biogenesis. Curr Biol. 2014;24:609–620. PMID:24613307. 24613307 10.1016/j.cub.2014.02.008 PMC4016984

[23] Kim J, Kundu M, Viollet B, et al. AMPK and mTOR regulate autophagy through direct phosphorylation of Ulk1. Nat Cell Biol. 2011;13:132–141. PMID:21258367. 21258367 10.1038/ncb2152 PMC3987946

[24] Kankotia S, Stacpoole PW. Dichloroacetate and cancer: new home for an orphan drug? Biochim Biophys Acta. 2014;1846:617–629. PMID:25157892. 25157892 10.1016/j.bbcan.2014.08.005

[25] Lin G, Hill DK, Andrejeva G, et al. Dichloroacetate induces autophagy in colorectal cancer cells and tumours. Br J Cancer. 2014;111:375–385. PMID:24892448. 24892448 10.1038/bjc.2014.281 PMC4102941

[26] Aronson LI, Davenport EL, Mirabella F, et al. Understanding the interplay between the proteasome pathway and autophagy in response to dual PI3K/mTOR inhibition in myeloma cells is essential for their effective clinical application. Leukemia. 2013;27:2397–2403. PMID:23670295. 23670295 10.1038/leu.2013.150 PMC3865535

[27] Fan Q-W, Cheng C, Hackett C, et al. Akt and autophagy cooperate to promote survival of drug-resistant glioma. Sci Signal. 2010;3:ra81. PMID:21062993. 21062993 10.1126/scisignal.2001017 PMC3001107

[28] Lin G, Andrejeva G, Wong Te Fong A-C, et al. Reduced Warburg effect in cancer cells undergoing autophagy: steady- state 1H-MRS and real-time hyperpolarized 13C-MRS studies. PLoS One. 2014;9:e92645. PMID:24667972. 24667972 10.1371/journal.pone.0092645 PMC3965444

[29] Zhang L, Yu J, Park BH, et al. Role of BAX in the apoptotic response to anticancer agents. Science. 2000;290:989–992. PMID:11062132. 11062132 10.1126/science.290.5493.989

[30] Shoji-Kawata S, Sumpter R, Leveno M, et al. Identification of a candidate therapeutic autophagy-inducing peptide. Nature. 2013;494:201–206. PMID:23364696. 23364696 10.1038/nature11866 PMC3788641

[31] Radhakrishnan A, Goldstein JL, McDonald JG, et al. Switch-like control of SREBP-2 transport triggered by small changes in ER cholesterol: a delicate balance. Cell Metab. 2008;8:512–521. 19041766 10.1016/j.cmet.2008.10.008 PMC2652870

[32] Mizushima N, Yoshimori T, Levine B. Methods in mammalian autophagy research. Cell. 2010;140:313–326. 20144757 10.1016/j.cell.2010.01.028 PMC2852113

[33] Jao CY, Roth M, Welti R, et al. Metabolic labeling and direct imaging of choline phospholipids in vivo. Proc Natl Acad Sci U S A. 2009;106:15332–15337. PMID:19706413. 19706413 10.1073/pnas.0907864106 PMC2741251

[34] Singh R, Kaushik S, Wang Y, et al. Autophagy regulates lipid metabolism. Nature. 2009;458:1131–1135. PMID:19339967. 19339967 10.1038/nature07976 PMC2676208

[35] Al-Saffar NMS, Jackson LE, Raynaud FI, et al. The phosphoinositide 3-kinase inhibitor PI-103 downregulates choline kinase alpha leading to phosphocholine and total choline decrease detected by magnetic resonance spectroscopy. Cancer Res. 2010;70:5507–5517. PMID:20551061. 20551061 10.1158/0008-5472.CAN-09-4476 PMC2896552

[36] Al-Saffar NMS, Marshall LV, Jackson LE, et al. Lactate and choline metabolites detected in vitro by nuclear magnetic resonance spectroscopy are potential metabolic biomarkers for PI3K inhibition in pediatric glioblastoma. PLoS One. 2014;9:e103835. PMID:25084455. 25084455 10.1371/journal.pone.0103835 PMC4118961

[37] Li Z, Vance DE. Phosphatidylcholine and choline homeostasis. J Lipid Res. 2008;49:1187–1194. PMID:18204095. 18204095 10.1194/jlr.R700019-JLR200

[38] Pelech SL, Vance DE. Regulation of phosphatidylcholine biosynthesis. Biochim Biophys Acta. 1984;779:217–251. PMID:6329299. 6329299 10.1016/0304-4157(84)90010-8

[39] Utal AK, Jamil H, Vance DE. Diacylglycerol signals the translocation of CTP: choline-phosphatecytidylyltransferase in HeLa cells treated with 12-O-tetradecanoylphorbol-13-acetate. J Biol Chem. 1991;266:24084–24091. PMID:1660890. 1660890

[40] Esko JD, Raetz CR. Autoradiographic detection of animal cell membrane mutants altered in phosphatidylcholine synthesis. Proc Natl Acad Sci U S A. 1980;77:5192–5196. PMID:6254065. 6254065 10.1073/pnas.77.9.5192 PMC350023

[41] Testerink N, van der Sanden MHM, Houweling M, et al. Depletion of phosphatidylcholine affects endoplasmic reticulum morphology and protein traffic at the Golgi complex. J Lipid Res. 2009;50:2182–2192. PMID:19458387. 19458387 10.1194/jlr.M800660-JLR200 PMC2759824

[42] Hoover-Fong J, Sobreira N, Jurgens J, et al. Mutations in PCYT1A, encoding a key regulator of phosphatidylcholine metabolism, cause spondylometaphyseal dysplasia with cone-rod dystrophy. Am J Hum Genet. 2014;94:105–112. PMID:24387990. 24387990 10.1016/j.ajhg.2013.11.018 PMC3882727

[43] Haider A, Wei Y-C, Lim K, et al. PCYT1A regulates phosphatidylcholine homeostasis from the inner nuclear membrane in response to membrane stored curvature elastic stress. Dev Cell. 2018;45:481–495.e8. 29754800 10.1016/j.devcel.2018.04.012 PMC5971203

[44] Lodish H, Berk A, Zipursky SL. Biomembranes: structural organization and basic functions [Internet]. In: Molecular cell biology. 4th ed. New York: W. H. Freeman; 2000. Section 5.3.

[45] Hatch GM, Oskin A, Vance DE. Involvement of the lysosome in the catabolism of intracellular lysophosphatidylcholine and evidence for distinct pools of lysophosphatidylcholine. J Lipid Res. 1993;34:1873–1881. PMID:8263412. 8263412

[46] Reunanen H, Hirsimäki P. Studies on vinblastine-induced autophagocytosis in mouse liver. IV. Origin of membranes. Histochemistry. 1983;79:59–67. PMID:6139354. 6139354 10.1007/BF00494342

[47] Ogasawara Y, Itakura E, Kono N, et al. Stearoyl-CoA desaturase 1 activity is required for autophagosome formation. J Biol Chem. 2014;289:23938–23950. PMID:25023287. 25023287 10.1074/jbc.M114.591065 PMC4156085

[48] Peck B, Schug ZT, Zhang Q, et al. Inhibition of fatty acid desaturation is detrimental to cancer cell survival in metabolically compromised environments. Cancer Metab. 2016;4:6. PMID:27042297. 27042297 10.1186/s40170-016-0146-8 PMC4818530

[49] Russo SB, Baicu CF, Van Laer A, et al. Ceramide synthase 5 mediates lipid-induced autophagy and hypertrophy in cardiomyocytes. J Clin Invest. 2012;122:3919–3930. PMID:23023704. 23023704 10.1172/JCI63888 PMC3484448

[50] Yamagata M, Obara K, Kihara A. Sphingolipid synthesis is involved in autophagy in Saccharomyces cerevisiae. Biochem Biophys Res Commun. 2011;410:786–791. PMID:21703229. 21703229 10.1016/j.bbrc.2011.06.061

[51] Corcelle-Termeau E, Vindeløv SD, Hämälistö S, et al. Excess sphingomyelin disturbs ATG9A trafficking and autophagosome closure. Autophagy. 2016;12:833–849. PMID:27070082. 27070082 10.1080/15548627.2016.1159378 PMC4854555

[52] Fengsrud M, Erichsen ES, Berg TO, et al. Ultrastructural characterization of the delimiting membranes of isolated autophagosomes and amphisomes by freeze-fracture electron microscopy. Eur J Cell Biol. 2000;79:871–882. PMID:11152279. 11152279 10.1078/0171-9335-00125

[53] Payne F, Lim K, Girousse A, et al. Mutations disrupting the Kennedy phosphatidylcholine pathway in humans with congenital lipodystrophy and fatty liver disease. Proc Natl Acad Sci U S A. 2014;111:8901–8906. PMID:24889630. 24889630 10.1073/pnas.1408523111 PMC4066527

[54] Gabellieri C, Beloueche-Babari M, Jamin Y, et al. Modulation of choline kinase activity in human cancer cells observed by dynamic 31P NMR. NMR Biomed. 2009;22:456–461. PMID:19156696. 19156696 10.1002/nbm.1361

[55] Tyagi RK, Azrad A, Degani H, et al. Simultaneous extraction of cellular lipids and water-soluble metabolites: evaluation by NMR spectroscopy. Magn Reson Med. 1996;35:194–200. PMID:8622583. 8622583 10.1002/mrm.1910350210

[56] Sze DY, Jardetzky O. Characterization of lipid composition in stimulated human lymphocytes by 1H-NMR. Biochim Biophys Acta Mol Cell Res. 1990;1054:198–206. 10.1016/0167-4889(90)90241-5 2400782

[57] Sitter B, Sonnewald U, Spraul M, et al. High-resolution magic angle spinning MRS of breast cancer tissue. NMR Biomed. 2002;15:327–337. PMID:12203224. 12203224 10.1002/nbm.775

[58] Dunn KW, Kamocka MM, McDonald JH. A practical guide to evaluating colocalization in biological microscopy. Am J Physiol Cell Physiol. 2011;300:C723–4. 21209361 10.1152/ajpcell.00462.2010 PMC3074624

[59] Manders EMM, Verbeek FJ, Aten JA. Measurement of co-localization of objects in dual-colour confocal images. J Microsc. 1993;169:375–382. 33930978 10.1111/j.1365-2818.1993.tb03313.x

[60] Zinchuk V, Zinchuk O, Okada T. Quantitative colocalization analysis of multicolor confocal immunofluorescence microscopy images: pushing pixels to explore biological phenomena. Acta Histochem Cytochem. 2007;40:101–111. 17898874 10.1267/ahc.07002 PMC1993886

[61] Stoica R, De Vos KJ, Paillusson S, et al. ER-mitochondria associations are regulated by the VAPB-PTPIP51 interaction and are disrupted by ALS/FTD-associated TDP-43. Nat Commun. 2014;5:3996. 24893131 10.1038/ncomms4996 PMC4046113

