# Supplementary material for: De novo phosphatidylcholine synthesis is required for autophagosome membrane formation and maintenance during autophagy
Source: Autophagy. 2019 Sep 13;16(6):1044–60. doi: 10.1080/15548627.2019.1659608 (PMC7469489; doi:10.1080/15548627.2019.1659608)
Supplement: Supplemental Material [file KAUP_A_1659608_SM8557.docx]

# Supplemental Material to:

# *De novo* phosphatidylcholine synthesis is required for autophagosome membrane formation and maintenance during autophagy

Gabriela Andrejeva^1+^, Sharon Gowan^2+^, Gigin Lin^1,3^, Anne-Christine LF Wong Te Fong^1^, Elham Shamsaei^1^, Harry G. Parkes^1^, James Mui^2^, Florence I. Raynaud^2^, Yasmin Asad^2^, Gema Vizcay-Barrena^4^, Joanna Nikitorowicz-Buniak^5^, Melanie Valenti^2^, Louise Howell^6^, Roland A. Fleck^4^, Lesley-Ann Martin^5^, Vladimir Kirkin^2^, Martin O. Leach^1,*^, Yuen-Li Chung^1,^^[[1]](#footnote-1)^

## Figure S1

| 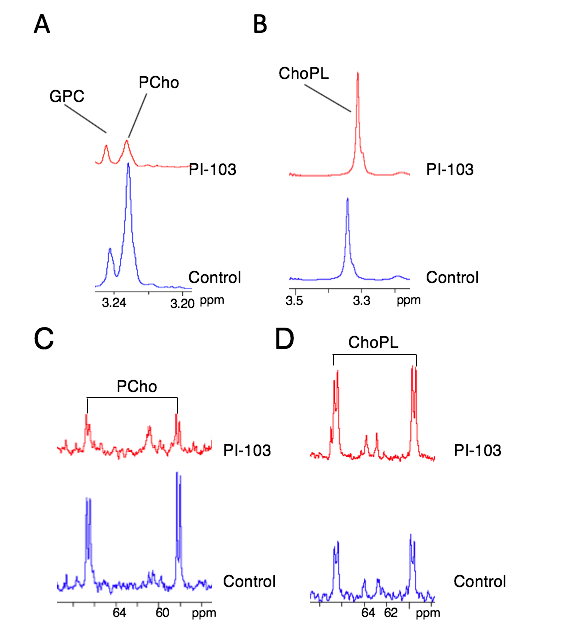 |
| --- |
| **Figure S1**. Examples of ^1^H- and ^13^C-MRS spectra of choline metabolites in cell extracts. Examples of ^1^H-MRS spectra of water-soluble (**A**) and lipid (**B**) phases showing phospholipid metabolites in DMSO vehicle- and 20 μM PI-103-treated HCT116 Bax-ko cells following 24 h of treatment for measurements in Fig. 1E. Examples of ^13^C-MRS spectra of water-soluble (**C**) and lipid (**D**) metabolite spectra in HCT116 Bax-ko DMSO control and 20 μM PI-103-treated cells that had [1,2-^13^C]choline medium added in the last 6 h of 24-h treatment for measurements in Fig. 1D. ChoPL: choline phospholipid; GPC: glycerophosphocholine; PCho: phosphocholine. |

## Figure S2

|   **Figure S2**. Time course of autophagy induction with DCA and PI-103 treatment in HCT116 *BAX*-ko cells. Western blots of autophagy marker LC3B in 6 h, 18 h and 24 h treatment with water vehicle-control, 75 mM DCA, DMSO-vehicle control or 20 μM PI-103 in HCT116 *BAX*-ko cells. TUBA was used as a loading control. |
| --- |

## Figure S3

**Figure S3.** Staining of Pro-ChoPLs and mCherry-LC3 in pMAFs cultured with or without Pro-Cho. (**A**) Imaging of mCherry-LC3 in mCherry-GFP-LC3 pMAF cells following 6 h of 250 nM Torin-1, 200 nM bafilomycin A_1_, 20 µM PI-103 or DMSO treatment. pMAF cells were treated for 6 h and then stained with Alexa Fluor 350-azide. (**B**) Imaging of Pro-ChoPLs and mCherry-LC3 in mCherry-GFP-LC3 pMAF cells following 6 h of 250 nM Torin-1, 200 nM bafilomycin A_1_, 20 µM PI-103 or DMSO treatment. pMAFs were treated the last 6 h before the end of 24 h incubation with Pro-Cho and then stained with Alexa Fluor 350-azide. The labeled Pro-ChoPLs signals (blue) were pseudo-colored to green using the software on the microscope, in order to enhance the contrast against the red signal. The focal yellow signals showing the colocalization of the Pro-ChoPLs and mCherry-LC3 stainings. Scale bars: 20 μm.

## Figure S4

|  |
| --- |

**Figure S4**. PCYT1A expression in autophagic cells. (**A**) Semi-quantification of PCYT1A expression in whole cell lysates normalized to TUBA. Data are expressed as mean ± SEM, normalized to unit 1 for the control treatment. N=6 for HCT116 *BAX*-ko PI-103, n=2 for HCT116 *BAX*-ko DCA and 6 h starved cells, n=1 for HT29 PI-103-treated cells. Statistically significant changes are indicated: *p<0.05, **p<0.01. (**B**) Western blots of PCYT1A in whole cell, soluble cytosolic and membrane fractions of 20 μM PI-103 (24 h)-treated HCT116 *BAX*-ko and in membrane fraction of 75 mM DCA (24 h)-treated and starved (6 h; in HBSS) HCT116 *BAX*-ko cells, and in 100 μM PI-103 (24 h)-treated HT29 cells. TUBA used as a soluble cytosolic protein and CTNNB1 as a membrane fraction protein loading controls and fractional enrichment markers.

## Figure S5

|   **Figure S5.** Cellular appearance of PI-103-treated cells with impaired PCYT1A activity. Light microscopy images of 18 h- and 24 h- DMSO-vehicle and 11.4 μM PI-103-treated CHO-K1 and MT58 cells at the permissive temperature of 33°C and the restrictive temperature of 40°C. Black arrows indicate potential autophagosomes or autolysosomes. |
| --- |

### Table S1. Summary of changes in cellular choline metabolites and cholesterol in drug- or starvation-induced autophagy models depicted in Fig. 1D.

|  | | | PCho | | GPC | | ChoPLs | | Cholesterol | |
| --- | --- | --- | --- | --- | --- | --- | --- | --- | --- | --- |
|  | | | fold change | p-value | fold change | p-value | fold change | p-value | fold change | p-value |
| HCT116  Bax-ko | PI-103 | 6 h | 0.62±0.10 | p=0.02 | 1.73±0.77 | p=0.81 | 1.28±0.13 | p=0.03 | 1.75±0.15 | p=8x10^-4^ |
|  |  | 24 h | 0.23±0.02 | p=2x10^-4^ | 1.19±0.11 | p=0.10 | 1.49±0.10 | p=0.006 | 1.42±0.10 | p=0.01 |
|  |  | 96 h | 0.36±0.03 | p=5x10^-4^ | 0.90±0.09 | p=0.40 | 1.91±0.07 | p=2x10^-4^ | 1.83±0.10 | p=6x10^-4^ |
|  |  | 19 h | 0.55±0.04 | p=0.002 | 0.73±0.08 | p=0.05 | 2.19±0.05 | p=1x10^-5^ | 1.74±0.10 | p=9x10^-4^ |
|  | DCA | 24 h | 0.32±0.02 | p=3x10^-7^ | 7.99±0.75 | p=4x10^-7^ | 1.11±0.02 | p=0.01 | 1.55±0.15 | p=2x10^-4^ |
|  | Starved | 6 h | 0.50±0.05 | p=3x10^-5^ | 1.13±0.81 | p=0.89 | 1.29±0.07 | p=0.003 | 1.35±0.15 | p=0.16 |
|  | Tat-Beclin 1 | 6 h | 0.68±0.02 | p=4x10^-6^ | nm | nm | 1.12±0.05 | p=0.03 | 1.08±0.08 | p=0.31 |
| HCT116  WT | DCA | 24 h | 0.12±0.02 | p=4x10^-6^ | 9.14±0.56 | p=1x10^-6^ | 1.31±0.04 | p=0.008 | 1.82±0.18 | p=9x10^-3^ |
|  | Starved | 6 h | 0.74±0.04 | p=0.05 | 0.35±0.15 | p=0.13 | 1.26±0.03 | p=1x10^-4^ | 1.18±0.21 | p=0.41 |
| HT29 | PI-103 | 24 h | 0.76±0.06 | p=0.04 | 0.77±0.14 | p=0.22 | 1.28±0.10 | p=0.02 | 1.54±0.21 | p=0.02 |

## Data shown are means of fold changes ± SEM, min *n*=3 in each group.

## Supplemental Materials and Methods

### *Digitoning permeabilization of cells for immunoblotting.*

For the separation of soluble and membrane proteins the cells were washed with PBS, permeabilized for 5 min in digitonin buffer (0.5 mg/ml digitonin; Thermo Scientific, BN2006), 0.25 mM sucrose (Fluka, 16104H), 10 mM Tris-HCl, pH 7.4 with protease inhibitor cocktail (Roche, 05892970001) [40]. The supernatant was collected for soluble protein fraction. The cells were then washed twice with PBS and lysed in RIPA buffer (Cell Signaling Technology, 9806) with protease inhibitor cocktail (Roche, 05892970001) for a minimum of 30 min on ice for the extraction of membrane fraction proteins. Both fractions were centrifuged at 16,000 *g* and the supernatants reserved for immunoblotting.

1. Correspondence: Dr Y-L Chung, E-mail: [ylichung@icr.ac.uk](mailto:ylichung@icr.ac.uk); or Professor MO Leach, E-mail: [martin.leach@icr.ac.uk](mailto:martin.leach@icr.ac.uk). [↑](#footnote-ref-1)
